# AlertGS: determining alerts for gene sets

**DOI:** 10.1093/bioinformatics/btaf133

**Published:** 2025-04-03

**Authors:** Franziska Kappenberg, Jörg Rahnenführer

**Affiliations:** Department of Statistics, TU Dortmund University, 44227 Dortmund, Germany; Department of Statistics, TU Dortmund University, 44227 Dortmund, Germany

## Abstract

**Motivation:**

A typical goal in gene expression studies is identifying certain gene sets enriched with significant genes. The measurement of many gene expression experiments for several concentrations or time points allows the modeling of the concentration/time–response relationship for each gene, and the subsequent estimation of a gene-wise alert. In this work, an approach is proposed to transfer the concept of alerts from single genes to gene sets, yielding a global significance statement and the respective concentration or time where the first enrichment of the gene set can be observed. The methodology is based on a Kolmogorov–Smirnoff type test statistic for each gene set.

**Results:**

Simulations show that a majority of these sets can be identified especially for lower numbers of true gene sets with a signal. The false positive rate can be controlled by subsequent decorrelation approaches. Overall, the true gene set-wise alerts are rarely overestimated and rather tend to be underestimated.

**Availability and implementation:**

The code needed to reproduce the simulations and apply the AlertGS methodology is available at the GitHub repository: https://github.com/FKappenberg/AlertGS.

## 1 Introduction

High-throughput technologies, e.g. DNA microarrays or RNA-Seq, allow the simultaneous measurement of tens of thousands of genes for different conditions, such as different times or the exposition with different concentrations of certain compounds. Typical analyses include comparing the gene expression for the exposed conditions to a negative control. Resulting lists of differentially expressed genes should not only be interpreted on the individual gene level. Instead, genes should also be summarized into functional groups (gene sets) and enrichment of these sets should be assessed (Subramanian *et al.* 2005, Alexa *et al.* 2006). Possible types of gene sets are gene ontology (GO) groups (Gene Ontology Consortium 2000), pathways summarized in databases such as the Kyoto Encyclopedia of Genes and Genomes (Kanehisa and Goto 2000) or the Reactome Knowledgebase (Jassal *et al.* 2020), or gene sets defined by transcription factors (Essaghir *et al.* 2010). Typical approaches for analyzing gene sets for overrepresentation or enrichment include *overrepresentation analyses*, e.g. set-wise Fisher tests based on some cutoff on the gene-wise effect level, and *functional class scoring* based on the ranking of the gene-wise effect level, see Khatri *et al.* (2012); Nguyen *et al.* (2019) for more details.

Often, toxicological experiments are conducted for a negative control and increasing concentrations/doses/times of an exposition. When analyzing such data, often the goal is to calculate a specific alert, i.e. the concentration/dose/time at which a pre-specified effect level is attained. Such alerts can be calculated based on the actually measured exposition levels, or interpolation via the fitting of a (parametric) model can take place. Typically, such model-based alerts should be preferred, see Jensen *et al.* (2019) for a recent overview of the methodological approaches. Several types of alerts have been proposed in the context of gene expression data (Kappenberg *et al.* 2021, Möllenhoff *et al.* 2023). In this work, the focus is on the absolute lowest effective concentration [ALEC (Jiang 2013, Kappenberg *et al.* 2021)], which is the lowest concentration where a fitted model attains a fixed change in gene expression in comparison to the negative control.

In this work, an approach is presented to combine the interpretation of gene-expression experiments on the level of gene sets with the calculation of alerts. Our new AlertGS methodology allows the calculation of alerts for sets of genes, based on individual gene-wise alerts. The methodology is an extension of the functional class scoring approach “gene set enrichment analysis,” where a Kolmogorov–Smirnoff test statistic on the ranked genes is calculated (Subramanian *et al.* 2005). Here, instead of ranking genes according to their effect level or *P*-value, the test statistic is based on the individual sorted alerts.

Eleven different methods to derive transcriptomic point of departures (tPoD) based on gene-wise alert concentrations are analyzed and compared in Farmahin *et al.* (2017). These approaches are based on gene sets, specifically on pathways, and result in a single tPoD per compound. In contrast, in our newly proposed method, the goal is to determine alerts for multiple gene sets simultaneously.

The remainder of this paper is structured as follows. The AlertGS methodology with all prerequisites, including the calculation of gene-wise alerts, is introduced. A case study dataset is briefly described for the methodology’s application. Since the gene sets of interest for this case study are GO groups, the gene ontology is explained. The case study is analyzed descriptively, and the setup of the simulation study, which heavily depends on the structure of the case study, is explained in detail. The simulation results are summarized, and finally, the methodology is also applied to the case study dataset.

## 2 Materials and methods

This section introduces the new methodology “AlertGS” (alerts for gene sets). The gene ontology is explained as the type of gene set considered, and the dataset for the case study is presented.

### 2.1 AlertGS

The basis for the AlertGS methodology is high-dimensional gene expression data for several increasing concentrations/doses of a compound, or across several time points, which allows the calculation of gene-wise alerts. In this context, an alert is given by any concentration/dose/time, where a pre-specified response is attained (Kappenberg *et al.* 2021). Since concentrations, doses, or times have equivalent mathematical properties as predictor variables, these terms can be used interchangeably. In the following, “condition value” is often used as a collective term.

The goal of the AlertGS methodology is to calculate an alert for an entire set of genes based on gene-wise alerts. The proposed methodology works independently of the specific choice of alert, but it is aimed at model-based alerts, i.e. alerts not only restricted to the measured condition values in a respective experiment but obtained by fitting a (parametric) model, thus allowing interpolation. The ALEC (absolute lowest effective concentration) is a model-based alert explicitly proposed in the context of gene expression experiments and is used as an alert in all analyses in this work (Jiang 2013, Kappenberg *et al.* 2021).

The first step for calculating model-based gene-wise alerts is to model the relationship between condition and response values. Any appropriate modeling technique is suitable. In this work, the two-step procedure MCP-Mod (Multiple Comparison Procedure and Modeling) is used. This procedure was originally introduced for clinical studies (Bretz *et al.* 2005), but was successfully applied to genomic concentration– and time–response data (Duda *et al.* 2022, Kappenberg *et al.* 2024). In the first step of the procedure, for each gene, it is tested whether the dose–response relationship can be better described by a model from a pre-specified set of candidate models than by a flat profile. The respective model parameters are estimated only for candidate models with a significant test result. Each gene’s final model (“winner model”) is selected based on an information criterion. Further details are given in Supplementary Material A.

Based on the fitted parametric models, gene-wise alerts are calculated. Here, the ALEC is considered. For a concentration x,x≥0 and a parametric model f(x,Φ) with parameter vector Φ, the ALEC is defined as that concentration, where a pre-specified effect level λ is attained, i.e. f(ALEC,Φ)=λ. The ALEC is determined via a grid search. The value of λ is specified based on biological considerations. Here, it is proposed to define it based on the response value of the winner model f(x,Φ) evaluated at the lowest tested concentration $x_{0}$, plus a reasonable change in gene expression, e.g. $\lambda =f(x_{0},\Phi )+{\;\text{log}\;}_{2}(1.5)$ (Kappenberg *et al.* 2021). This definition assumes an up-regulation of the respective gene. An equivalent calculation in case of a down-regulated gene is given by $\lambda =f(x_{0},\Phi )-{\;\text{log}\;}_{2}(1.5)$.

For the procedure, it is assumed that only one direction (e.g. up-regulation) is of interest. However, the alerts in the respective “other direction” play a role in the calculation of the group-wise alert as well. In addition, it is generally not possible to calculate an alert for all genes, e.g. because the fitted model’s response range is not large enough. Within the AlertGS methodology, a procedure to still include genes with missing alerts is proposed.

**Figure 1.**
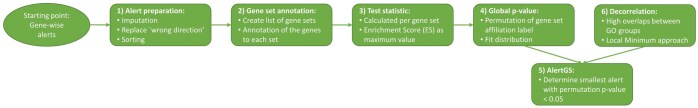
Flowchart summarizing all six steps of the AlertGS methodology, starting from gene-wise alerts.

The AlertGS procedure, starting from gene-wise alerts, is summarized in Fig. 1. The procedure consists of six steps: Preparing the gene-wise alerts, assigning the genes to the gene sets, calculating the running sum test statistic for each gene set, determining the global *P*-value, calculating the group-wise alert, and, if applicable, performing a decorrelation approach.

For the preparation of the gene-wise alerts, the alerts in the intended direction (i.e. up- or down-regulation) are sorted increasingly. Ties, i.e. two genes with the same alert, are avoided by adding a very small random number to duplicate values. By design of the experiment, there is a maximal tested condition value, denoted as $c_{\max}$, which is also the maximal possible value an alert can take. The idea now is to sort all genes increasingly, starting with the genes with alerts in the “correct direction,” then all genes without any alerts, and then all genes with alerts in the “wrong direction” reversed. This is achieved by assigning imputed alerts outside the range of observable values to genes without an estimated alert. Specifically, imputed alerts are sampled uniformly from the interval $\left[c_{\max}+\varepsilon ,2c_{\max}-\varepsilon \right]$ for an ε>0, and if a gene has an alert but in the “wrong direction,” the value is imputed by $3c_{\max}-$ alert.

In the second step, the gene sets of interest need to be defined. Specific annotation information is used depending on the organism assessed in the gene expression study.

The gene set-wise test statistics, global *P*-values, and alerts are calculated in Steps 3–5. The procedure here is analogous to the gene set enrichment analysis as proposed by Mootha *et al.* (2003) and Subramanian *et al.* (2005), but instead of considering ranks of genes, e.g. obtained by a sorted list of *P*-values, the alerts of the genes are used as basis. Consider a dataset consisting of N genes, all with prepared alerts as described before. Denote by M the size of the gene set under consideration, i.e. M of the alerts are labeled as “in the group” and N−M alerts are labeled as “not in the group.” The test statistic for the AlertGS is of a Kolmogorov–Smirnoff type, i.e. it is a running sum based on the sorted alert values of:

$$\{\begin{array}{cc} \sqrt{\frac{N-M}{M}} & \text{if}\;\text{gene}\;\text{is}\;\text{in}\;\text{the}\;\text{group}\\ -\sqrt{\frac{M}{N-M}} & \text{if}\;\text{gene}\;\text{is}\;\text{not}\;\text{in}\;\text{the}\;\text{group} \end{array}$$

This running sum starts and ends at zero, but takes positive or negative values throughout the alerts.

For the calculation of the global *P*-value, the maximum value of the test statistic in the interval $\left[0,c_{\max}\right]$ is determined. Following Mootha *et al.* (2003), this maximum value is denoted as Enrichment Score (ES). To determine the global significance, the labels (“in the group” and “not in the group”) are randomly permuted 1000 times, and for each permutation i, the respective Enrichment Score ES${}_{i}$ is calculated. The global *P*-value $\tilde{p}$ is determined as

(1) $$\tilde{p}=\frac{\sum_{i=1}^{1000}1_{\left\{ES_{i}\geq ES\right\}}}{1000}.$$

A low global *P*-value of a gene group indicates that the alerts of this group are smaller than those of all other genes.

A disadvantage of permutation *P*-values is the granularity of the results, as the number of permutations that can reasonably be evaluated is limited by computational power. Another disadvantage is that a *P*-value of exactly zero can occur in the case of extreme values for ES. With values of exactly zero, adjustment procedures and comparability of gene sets are limited. Thus, a density is fitted to all permutation-based values ES${}_{i}$ and the *P*-values are calculated as quantiles of this density. The values ES${}_{i}$ are nonnegative, their distribution is right-skewed with inflation of zeros, and extreme values can occur. To deal with the positiveness and the inflation of zero, a rectified distribution where all negative values are set to zero is chosen. Due to the skewness and the possible extreme values, a Gumbel distribution is used. The Gumbel distribution has a location parameter μ and a scale parameter β>0. Let X be a random variable following a Gumbel(μ, β) distribution, then $X^{+}=\max(0,X)$ follows a rectified Gumbel distribution. The parameters are estimated via a numerical optimization approach. The gene set-wise *P*-value based on the rectified Gumbel distribution is given by 1 minus the cumulative distribution function at ES.

Classic adjustment approaches for multiple testing [e.g. controlling the false discovery rate (Benjamini and Hochberg 1995)] can be applied to the global *P*-values. However, due to potential overlaps between gene sets, in some applications, this may introduce biases (Alexa *et al.* 2006). Thus, a specific decorrelation approach is proposed below.

The fifth step of the AlertGS procedure is calculating the gene set-wise alert, also denoted as AlertGS. This is based on the permutation test statistics, but only relevant for groups with a significant global *P*-value (if applicable, after adjusting for multiple testing, or performing decorrelation approaches). The gene-wise alerts are assessed in increasing order. For each alert a of a gene contained in the gene set, the value of the original test statistic at that value is evaluated and denoted as $RS^{a}$. The respective values of the permutation running sum test statistics at alert a are also assessed and denoted as $RS_{i}^{a}$ for i=1,…,1000. For alert a, the ratio $\frac{\sum_{i=1}^{1000}1_{\left\{RS_{i}^{a}\geq RS^{a}\right\}}}{1000}$ is calculated, and the lowest value a where this ratio takes a value of 0.05 or smaller is the resulting gene set-wise alert.

A detailed graphical example-based summary of the entire AlertGS procedure is shown in Supplementary Material A.

Especially the hierarchical structure of the gene ontology (see below) leads to gene sets with relevant overlap. Thus, a decorrelation approach for this type of gene set is proposed, named the “local minimum” (LocMin) approach. Within the hierarchical ordering of the gene sets, iteratively for each set, it is checked whether the global *P*-value is smaller than all *P*-values of parents and children. Only if this is the case, the *P*-value is retained, otherwise, it is not considered to be significant, independent of its actual value. This approach can be considered as a method for adjustment for multiple testing.

### 2.2 Case study: western diet mice

The data used for this case study is a follow-up experiment to the previously published data from Ghallab *et al.* (2021). In the data presented here, 90 mice were studied over a period of up to 48 weeks. Forty-five mice were fed a healthy “standard diet” (SD), and 45 received an unhealthy “western diet” (WD) but without trans fats. At Weeks 3, 6, 12, 18, 24, 30, 36, 42, and 48, five mice were sacrificed each; and among other measurements, gene expression from 35 727 genes was measured using RNA-seq. Further filtering and pre-processing are explained in Section 3.1.

### 2.3 Gene ontology

In the case study presented here, gene sets are defined by gene ontology (GO) groups. There are three GOs, with groups defined by biological processes, molecular functions, or cellular components of genes (Gene Ontology Consortium 2000). Here, only biological processes are considered. A GO is a directed acyclic graph with a hierarchical structure, where each node corresponds to a GO group. Nodes on top of the graph are high-level processes, and nodes further down in the graph are more specific. All genes annotated to a specific group are also contained in the respective parent group. A GO group may have several children and/or several parents.

## 3 Results

In this section, the results of the analyses are shown. Since the setup of the simulation study heavily depends on the structure of the case study, first a descriptive analysis of this dataset is presented, and then the setup of the simulation study is explained. Results from the simulation and applying the AlertGS methodology to the dataset are presented in detail. All analyses were conducted using the statistical software R [version 4.3.2 (R Core Team 2023), https://www.R-project.org/], with additional software packages as indicated in the respective parts of the manuscript.

### 3.1 Descriptive analysis of the case study

The dataset consists of 90 samples (45 mice fed WD, 45 mice fed SD) and 35727 variables (genes). First, the dataset was filtered for genes with a nonzero count in enough samples. Specifically, only those 15384 genes were retained where nonzero counts were observed in more than half, i.e. at least 23, of the samples for mice fed WD and SD, respectively. In the results presented here, only mice fed WD are considered. The software package DESeq2 [version 1.42 (Love *et al.* 2014)] was used for analyzing the dataset. A plot of the first two principal components of the 500 genes with the highest variability after performing a variance stabilizing transformation for the WD mice is shown in Supplementary Material C.

**Figure 2.**
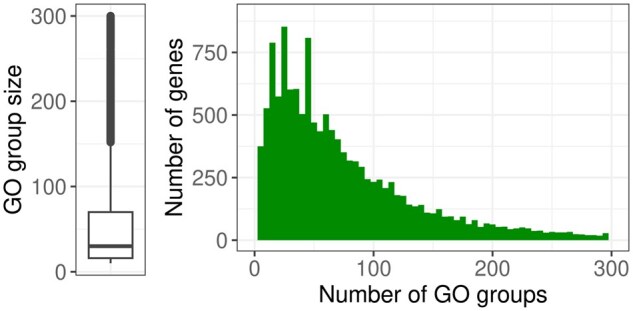
Left: Boxplot summarizing the sizes of the GO groups, 640 groups with sizes >300 not shown. Right: Histogram showing the number of GO groups (*x*-axis) to which each gene (*y*-axis) is annotated, 469 genes that are annotated to >300 groups are not shown.

To create a list of GO groups based on the measured genes, the annotation packages AnnotationDbi [version 1.60.0, (Pagès *et al.* 2022)] and org. Mm.eg.db [version 3.16.0, (Carlson 2022)] were used. Of the 15834 genes obtained after filtering 15188 are also included in the annotation package. GO groups from the ontology “biological processes” with at least 10 annotated genes were considered, resulting in 6447 groups. A boxplot summarizing the sizes of the resulting GO groups and a histogram showing the number of GO groups to which each gene is annotated are shown in Fig. 2.

### 3.2 Setup of the simulation study

The quality of the AlertGS methodology is assessed in a controlled simulation study. To take the complex correlations of gene sets into account, the case study is used as a framework for the simulation setup. The simulation takes place on the level of the gene-wise alerts, here, values between 3 and 48 (minimum and maximum time point of the underlying case study).

A null situation, in which no GO group is simulated to have an alert, is obtained by randomly sampling a certain percentage r of all genes. Random alerts for these genes are simulated by drawing from a uniform distribution on the interval [3,48]. The used percentages are 5%, 10%, and 15%.

In the alternative situations, some GO groups are simulated with group-wise alerts. These group-wise alerts form the basis to simulate the gene-wise alerts. Two procedures are identified: The first procedure considers the GO groups and their respective alerts to be independent of each other, the approach is thus denoted *independent*. In the second procedure, the groups are assessed iteratively in increasing order of their respective alerts. This approach is denoted as *iterative*. Both approaches are explained in detail in the following paragraphs. The groups simulated to have group-wise alerts are denoted as “meaningful groups” and are randomly selected among all groups with sizes between 20 and 200. Depending on the specific approach, a certain number of genes are chosen for a meaningful group to obtain simulated alerts (“meaningful alerts”) based on the group-wise alert. For a group with group-wise alert g, the meaningful alert of a gene is sampled from a shifted and scaled Beta distribution B(α,β)·scale+shift, with parameters α=β=2, scale =4 and shift =g−2. This results in a density function with support [g−2,g+2] and mean g. A plot of the density is shown in Supplementary Material B.

For both approaches, as a first step, integer-valued alerts g∈{5,6,…,46} are randomly drawn for the meaningful groups. A ratio x of genes with meaningful alerts is fixed for the entire simulation run.

In the independent approach, the meaningful groups are considered in the order they were drawn. For the first meaningful group, x times the group size (rounded up) genes are selected and meaningful alerts are drawn for these genes. Due to high overlaps between groups, for some genes, there may already be an alert for the second group and onwards. Thus, genes are only selected from those genes that do not already have an alert. In cases where not enough genes without alert are available, all remaining genes without alert are chosen as meaningful genes.

In the iterative approach, the meaningful groups are sorted increasingly by their alerts. Starting with the group with the smallest alert g, x times the group size (rounded up) genes are selected and meaningful alerts are drawn for these genes. Iteratively, the group with the next largest alert is chosen, and x times the group size (rounded up) genes are randomly sampled from all genes in the group. Only for those genes that do not yet have a simulated alert, a meaningful alert is randomly drawn. This approach follows the biological motivation that a gene might be part of several biological processes. If it obtains meaningful alerts from more than one group, the final alert should come from the group with the smallest value.

In both approaches, as a last step, a certain ratio of genes is sampled from all genes without considering their group annotation. For these genes, an alert is sampled uniformly on the interval [3,48]. However, this value is only retained if the gene does not have a previously sampled meaningful alert that is smaller than the here-sampled value. A graphical display of the entire procedure is given in Supplementary Material B.

Scenarios for the independent and iterative approach are obtained by varying the number of meaningful groups n (values: 10, 20, 50) and the ratio of genes with meaningful alerts x (values: 0.1, 0.3, 0.5). Table 1 gives an overview of all scenarios. The simulation is conducted with 200 runs per scenario.

**Table 1.** Summary of all simulation scenarios, where for the two alternative situations, all possible combinations of numbers of meaningful groups and ratios of meaningful genes are considered.

	Null Sit.	Alternative Sit.	
		Independent	Iterative
Nr mean. groups	0	10, 20, 50	10, 20, 50
Ratio mean. genes	N/A	0.1, 0.3, 0.5	0.1, 0.3, 0.5
Ratio unif. genes	0.05, 0.1, 0.15	0.1	0.1

### 3.3 Results of simulation study

The simulation results presented in this section always refer to the LocMin approach, where a global *P*-value for a group is only retained if the *P*-value is the respective minimum of the group and its parents and children. Corresponding plots for the unadjusted *P*-values are given in Supplementary Material C.

**Figure 3.**
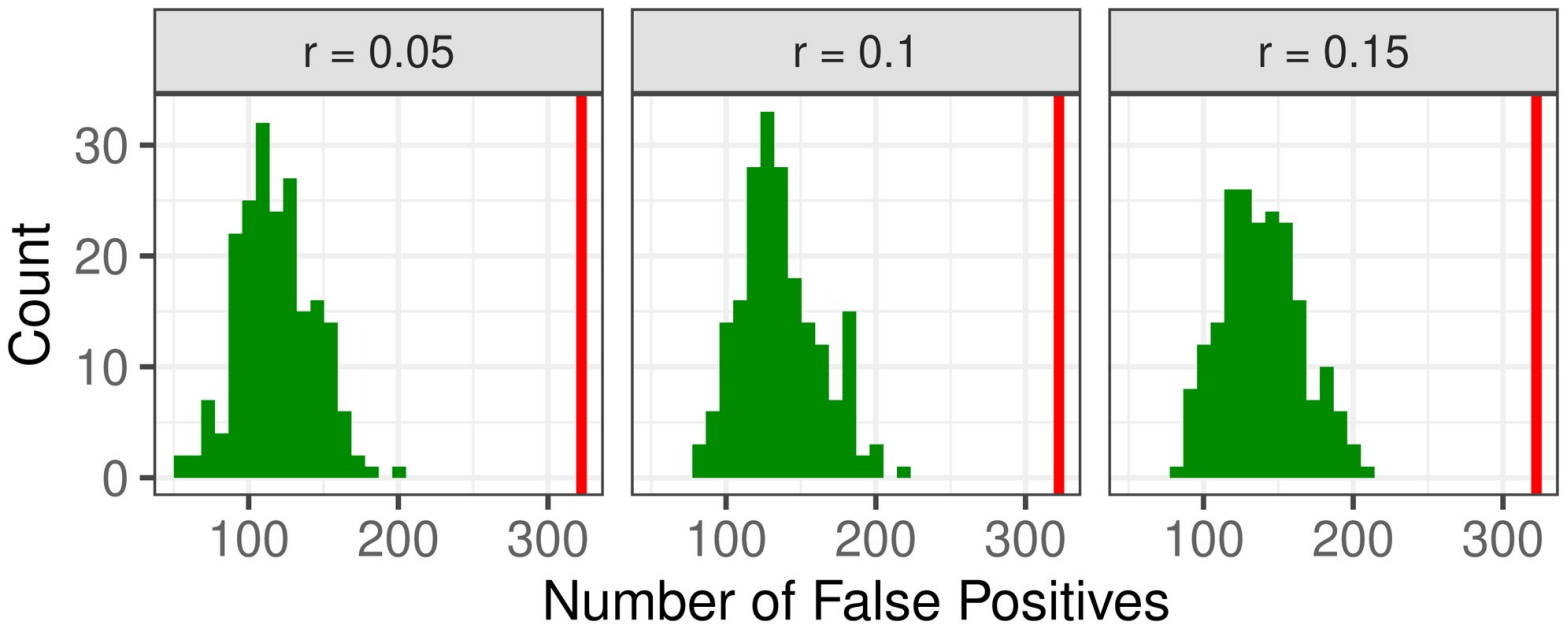
Number of false positive GO groups in the null situation with three different percentages of genes with uniformly sampled alerts. The vertical line indicates the level of 5%.

First, the null situation without meaningful groups, but with a certain ratio r of uniformly sampled alerts is considered. The numbers of false positives, i.e. groups with global *P*-value $\tilde{p}<0.05$, across 200 simulation runs, for three scenarios, are shown in Fig. 3. The total number of groups is 6447, and the level of 5%, corresponding to 322 groups, is indicated by a vertical line.

**Figure 4.**
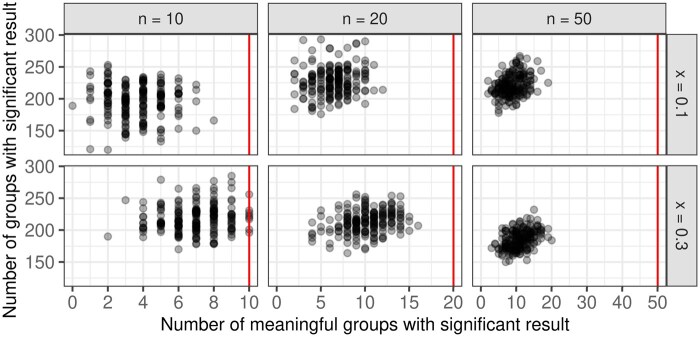
Summary of the number of true positives, i.e. numbers of meaningful groups with significant results, and the overall number of groups with a significant result, for all simulation scenarios. The number of true positives is bounded by the number of meaningful groups in the respective scenario, this is indicated by the vertical line (red).

For the alternative situation, two simulation approaches, namely the independent and the iterative approach, are considered. Since the iterative approach follows more closely the biological interpretation of processes starting in a sequence one after the other, results from this approach are presented here. Results from the independent approach are shown in Supplementary Material C, as well as the results for x=0.5 since they are very similar to the results for x=0.3. Figure 4 summarizes the respective number of meaningful groups with significant results, and the overall number of groups with significant results (i.e. true positives and false positives) for all simulation scenarios. Most true positives are reliably identified, especially for lower numbers of true positives. At the same time, the total number of identified groups decreases with more meaningful groups.

**Figure 5.**
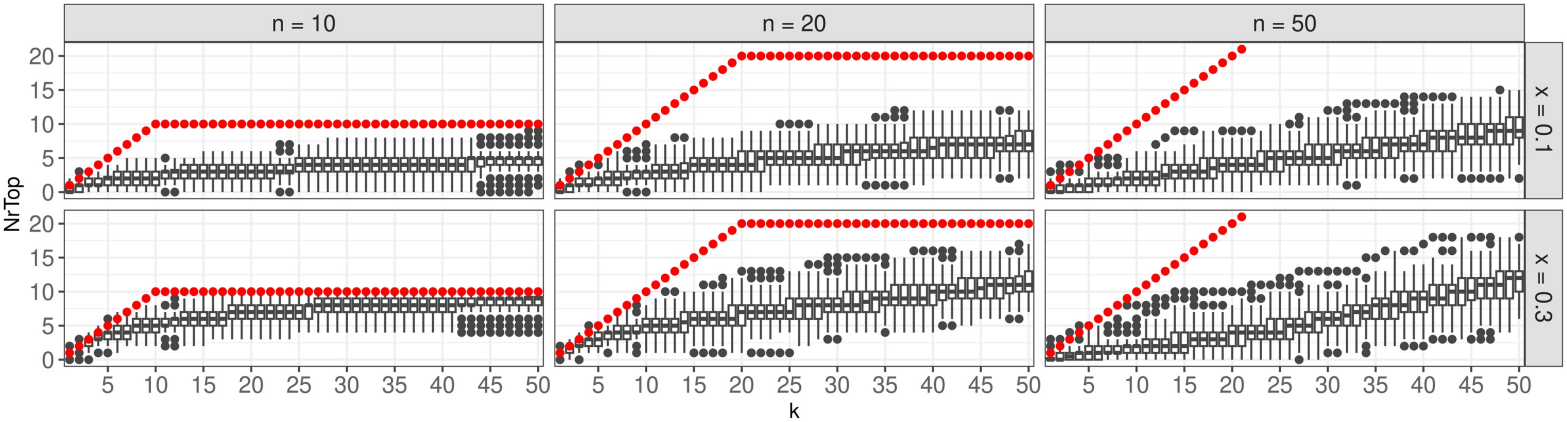
Boxplots showing for k=1,…,50 how many of the top-*k* identified groups are meaningful groups or the parent/child of a true meaningful group. The true number of meaningful groups is indicated by red dots. Observed numbers larger than the respective *k* might occur due to the additional consideration of the parents and children of the true groups.

Due to the hierarchical structure of the GO graph, large overlaps between GO groups occur. Applying the LocMin approach reduces the overall number of identified GO groups, however, this may lead to cases where not an actual meaningful group is identified as significant, but its parent or child. Still, such a scenario could be considered as a correctly identified group, since similarities between parents and children groups are very high. In the next analysis, thus, this knowledge is taken into account. Figure 5 shows boxplots of the corresponding correctly identified meaningful groups, or their parents or children, among the respective top-k groups with the smallest global *P*-values. Specifically, all groups are sorted increasingly according to their global *P*-value $\tilde{p}$ after applying the LocMin condition. Iteratively, for k=1,…,50, the top-k identified groups are assessed. For each meaningful group, it is checked whether the group itself or one of its parents/children is contained in the top-k identified groups. The number of meaningful groups for which this is the case is counted.

**Figure 6.**
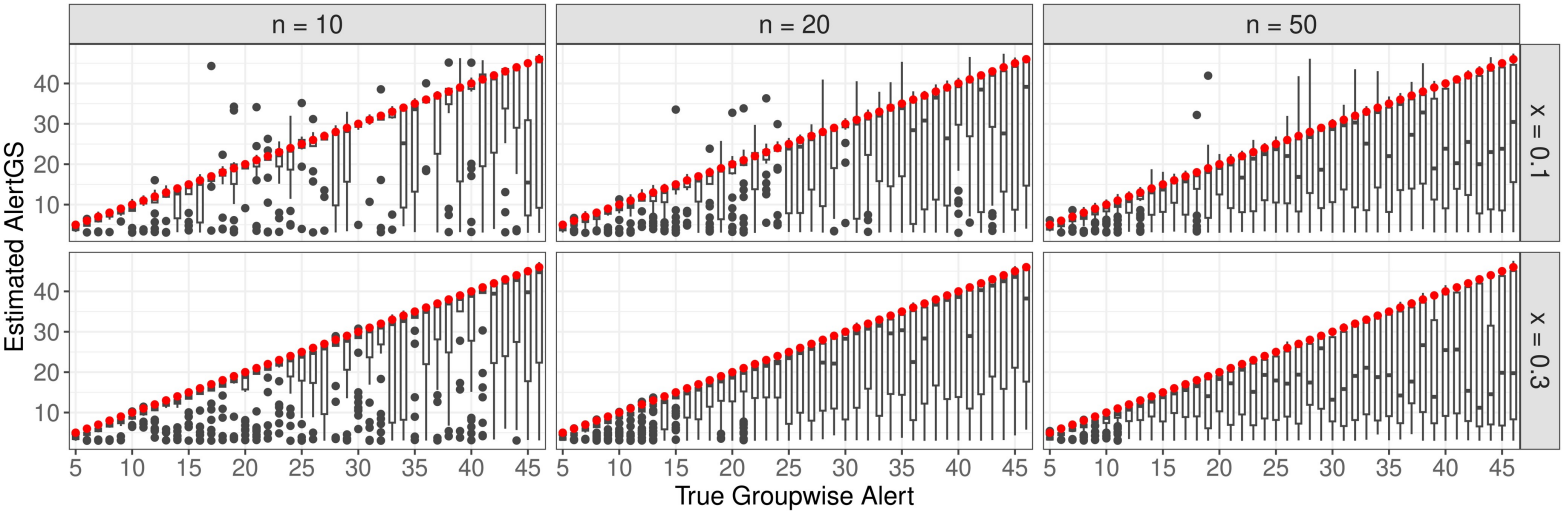
Comparison of the estimated AlertGS values and the respective true group-wise alerts (red dots) for the correctly identified significant groups.

Finally, the estimated AlertGS values for the correctly identified groups are plotted on the *y*-axis against the respective true group-wise alerts on the *x*-axis, which are additionally indicated by the red dots (Fig. 6). Overall, only a few cases with too large estimates can be observed, whereas there is a tendency to underestimate the true alerts, especially for larger true values of the AlertGS.

In addition to comparing the results of the controlled simulation study to the underlying truth, a comparison with the conventional approach of determining significant GO groups for pre-specified time cutoffs is performed. The approach is as follows. A time-cutoff is determined, here set to a value of 20. Based on the individual gene-wise alerts and this cutoff, Fisher-test-based GO overrepresentation analysis is conducted. The details of this method are explained in Supplementary Material A. The software package topGO [version 2.50.0 (Alexa *et al.* 2006)] was used. To take the correlation structure of the GO groups into account, the specific decorrelation approach elim is used. In this approach, the GO groups are assessed bottom-up in the hierarchy, and genes from significant groups are removed from further analyses. A comparison of the groups identified by this approach with the true meaningful groups is given in Supplementary Material C, showing true positive rates close to 1, but especially for higher number of meaningful groups also high numbers of false positives. The identified groups are then compared to groups with an AlertGS value smaller than or equal to 20, in the LocMin approach. Since the different decorrelation approaches can lead to slightly different identified groups, e.g. by finding parents or children, a direct comparison only of the identified groups by both approaches might lead to negatively biased results.

**Figure 7.**
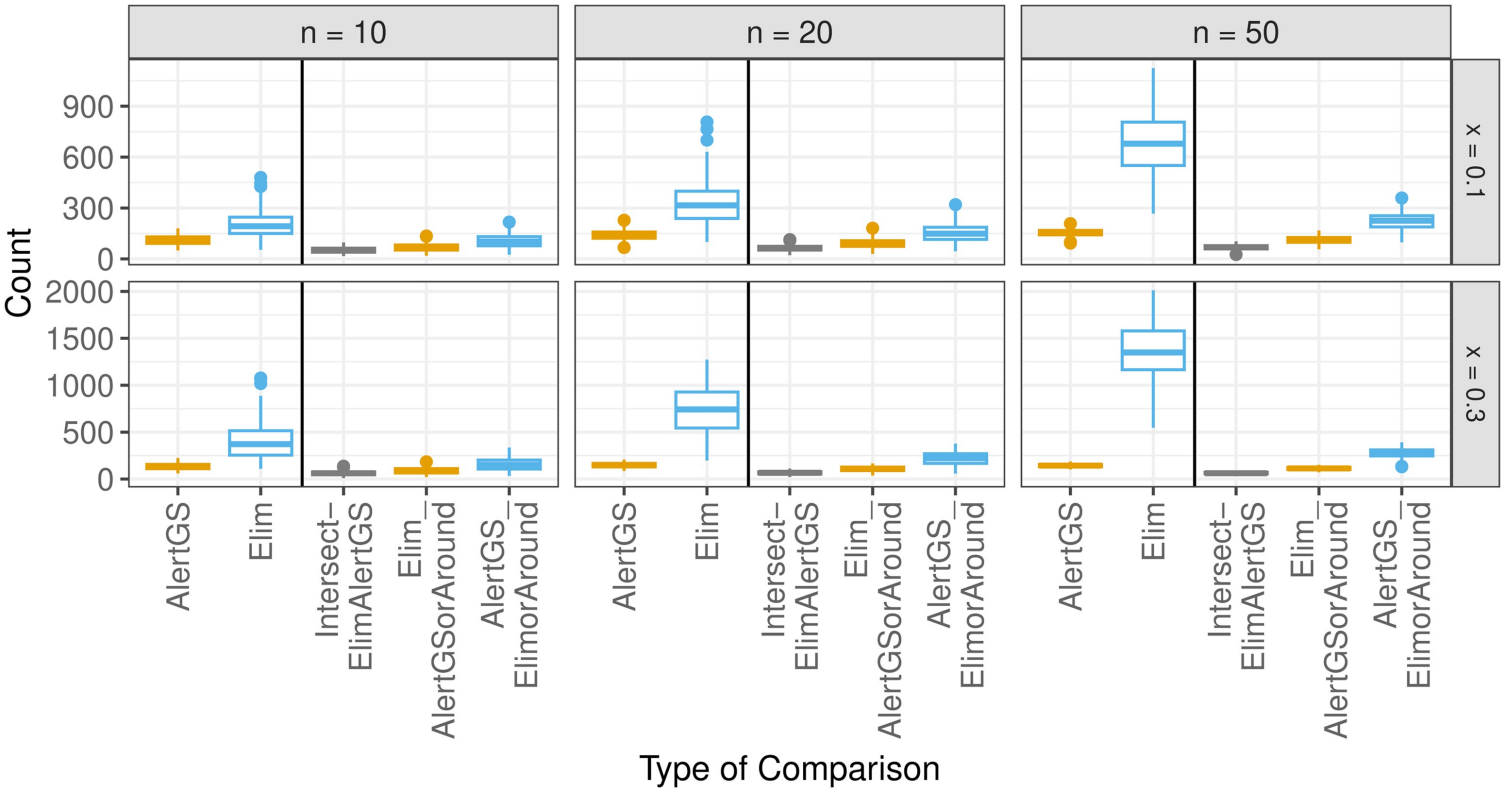
Boxplots indicating the numbers of identified groups with the AlertGS and the Elim approach, as well as the number of intersections between these lists, the number of groups found by the AlertGS approach, where either the group itself or a parent/child of the group is also found by the Elim approach, and vice versa.

Here, similar to the analyses shown in Fig. 5, this is considered. In addition to comparing the resulting lists of GO groups, the respective comparisons also taking parents and children into account are performed. Figure 7 shows boxplots of the respective numbers of identified GO groups with the Fisher-based “Elim” approach, and the AlertGS approach across all 200 simulation runs. In addition to the number of groups contained in both resulting lists of significant groups (“Intersect-ElimAlertGS”), the number of groups found by the AlertGS approach, where either the group itself or a parent/child of the group is also found by the Elim approach (“Elim_AlertGSorAround”) or vice versa (“AlertGS_ElimorAround”) are displayed. The colors are chosen for better interpretability: The maximum observable number of “Elim_AlertGSorAround” in each simulation run is given by the respective number of “AlertGS,” so these are both colored in orange, and analogously, “AlertGS_ElimorAround” and “Elim” are both colored in blue. The “Elim” approach identifies more significant groups than the AlertGS approach. While the number of groups found by both approaches (“Intersect-ElimAlertGS”) is very low, the comparisons taking parents/children into account are higher, particularly for the comparison of the groups found by AlertGS to the groups found by Elim, or their parents/children (orange boxes). A version of this graphic with a smaller *y*-axis is shown in Supplementary Material C.

### 3.4 Case study

After filtering and pre-processing the case study dataset as described in Section 3.1, 15188 genes are considered for model-fitting. The MCP-Mod methodology is applied to all genes, with linear, quadratic, exponential, emax, sigEmax, and beta model as candidate models. The specific parameter guesstimates and corresponding candidate model shapes are chosen as shown in the detailed explanation of the methodology in Supplementary Material A. The software package DoseFinding [version 1.1–1 (Bornkamp *et al.* 2023)] is used. For 10 160 genes, a signifMCP is established, and for 1475 genes, an ALEC for upregulation, and for 618 genes, an ALEC for downregulation can be calculated. A complete overview of the respective numbers of genes and alerts is given in Supplementary Material C. The number of times each of the six models is the winner model is summarized in Table 2. More information on the winner models is given in Supplementary Material C.

**Table 2.** Number of times each of the six candidate models is the winner model in the MCP-Mod methodology.

Linear	Quadratic	Exponential	emax	sigEmax	betaMod
2717	1417	442	3193	1357	1034

**Figure 8.**
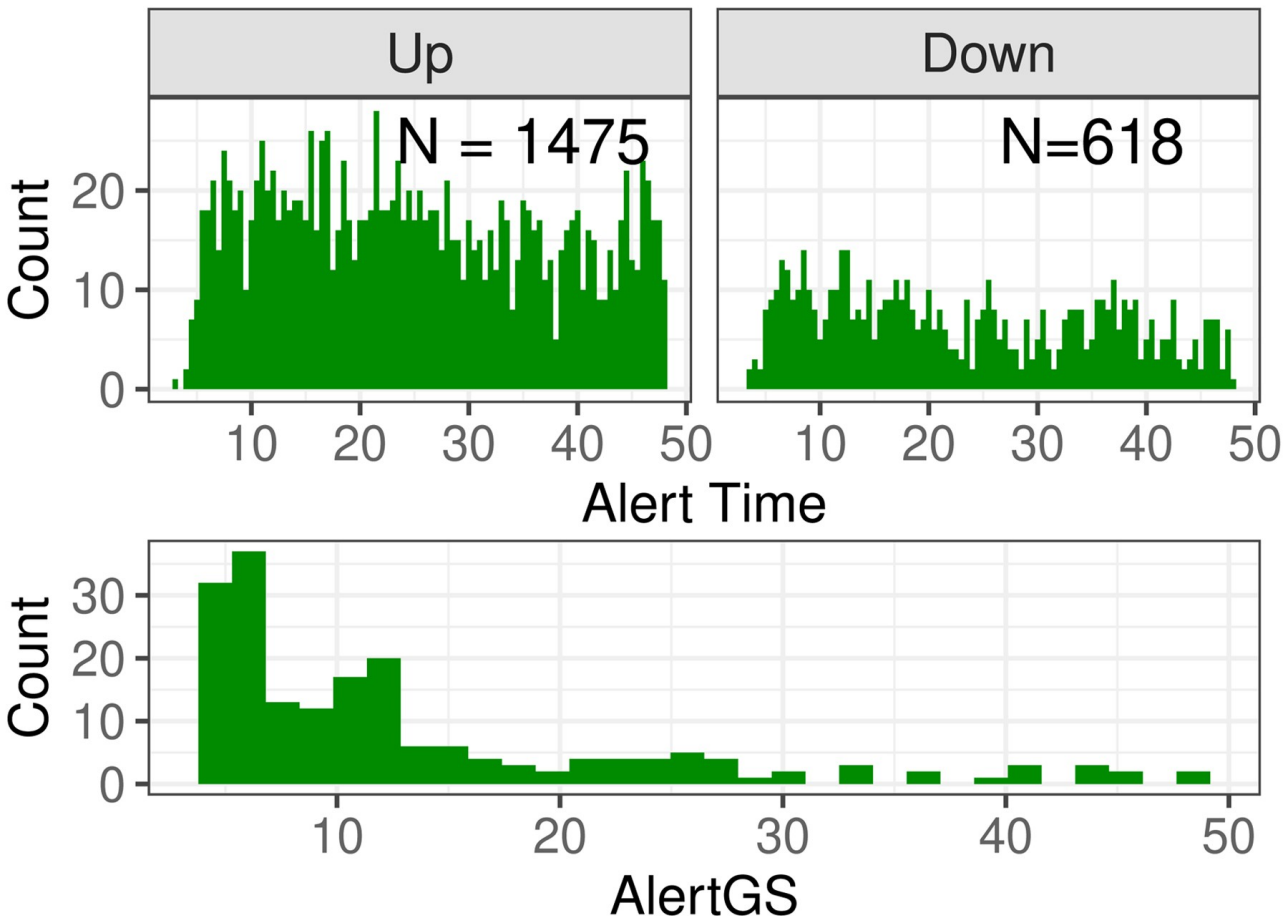
Top: Calculated alert times for the WD mice dataset, divided into up- and downregulated genes. Bottom: AlertGS for the 192 groups with $\tilde{p}<0.05$ and fulfilling the LocMin condition.

Based on the fitted parametric curves, ALEC values are calculated. If for some genes due to a strong nonmonotonous shape both an ALEC in up- and downregulated direction can be calculated, only the minimum value is retained. The distribution of the ALEC values is shown in Fig. 8.

After preparation of the alerts of all genes as described before, the AlertGS approach is applied to each GO group, yielding a global *P*-value, and, where applicable, an estimate of the group-wise alert. Of all 6447 considered GO groups, 2439 have a global *P*-value $\tilde{p}<0.05$. When following the LocMin approach, 192 groups have a global *P*-value $\tilde{p}<0.05$ and are the local minimum compared to their parents and children. The five groups with the smallest global *P*-value fulfilling the LocMin condition are summarized in Table 3. The complete results for all 6447 groups are given in an interactive supplementary table in Supplementary Material D.

**Table 3.** Information on the five GO groups with smallest *P*-values fulfilling the LocMin condition.^a^

Group ID	Annot.	Alerts Gr.	ArgMax	AlertGS	PVal
GO:0009605	2094	449	47.60	4.81	.00
GO:0009607	1162	297	47.60	5.65	.00
GO:0001775	948	246	47.69	5.59	.00
GO:0007155	1203	278	47.86	5.09	.00
GO:0040011	1154	266	47.38	5.73	.00

a Annot. states the number of genes, Alerts Gr. states the number of genes with alert, ArgMax gives the time where the test statistic is maximal, AlertGS is the group-wise alert, and PVal states the *P*-value, here always rounded to a value of 0.

A histogram of the resulting AlertGS values for groups with $\tilde{p}<0.05$, fulfilling the LocMin condition, is shown in Fig. 8. The histogram for all groups with $\tilde{p}<0.05$, but not necessarily fulfillment of the LocMin condition, is shown in Supplementary Material C.

A comparison of the results obtained with AlertGS and from the Fisher-test-based Elim approach with a cutoff of 20 weeks for the case study data is shown in Supplementary Material C. The results show generally good agreement between the groups identified with the two approaches, with many identified groups with the Elim approach.

## 4 Conclusion

This work proposes a method to transfer the concept of alerts, i.e. the concentration or time at which a pre-specified effect is attained, from individual genes to entire sets of genes. This results in information on whether more genes than expected at random assigned to a specific gene set have an alert (global *P*-value) and the respective alert (time or concentration) where the gene set first shows activation. The approach is based on calculating a Kolmogorov–Smirnoff type test statistic for each gene set based on the individual gene-wise alerts. This method and the resulting estimate are denoted by “AlertGS.” In simulations and comparison to established approaches based on fixed cutoffs, results showed a reasonable rate of correctly identified GO groups, with a tendency to underestimate the true gene set-wise alerts. In most toxicological applications, this is preferred to overestimation when determining the potential hazard of compounds. Possible extensions of the presented methodology include inserting a weight term for each gene, similar to the enrichment score originally introduced by Subramanian *et al.* (2005), where the correlation between the expression profile and the profile of interest is included in the test statistic. Here, the weight term can, e.g. be derived from the goodness of fit of the modeled concentration–response curve.

## Supplementary Material

btaf133_Supplementary_Data

## Data Availability

Simulation data are available via the code provided on GitHub.
