# Supplementary material for: AlertGS: determining alerts for gene sets
Source: Bioinformatics. 2025 Apr 3;41(4):btaf133. doi: 10.1093/bioinformatics/btaf133 (PMC12041417; doi:10.1093/bioinformatics/btaf133)
Supplement: btaf133_Supplementary_Data [file btaf133_supplementary_data.zip › AlertGS_SupplementD.html]

SupplementD.knit


# D Supporting Information for `AlertGS: Determining alerts for gene sets’

## Complete Results Table

The following table comprises all results from the AlertGS
methodology for all 6447 GO groups of size at least 10 that can be found
for the underlying data set. The table contains the following
information:

- GOId: Identifier of the GO group
- GOTerm: Description of the respective biological process
- Annotated: Number of genes in the GO group
- NrAlertGroup: Number of genes with an alert in the GO group
- ArgMax: X-axis value (here: time) at which the maximum of the test
  statistic is observed
- AlertGS: Group-wise alert, here displayed only for those groups with
  a global p-value <= 0.05
- GumbelPVal: Global p-value
- LocMinGumbel: Yes/No, whether the global p-value fulfills the LocMin
  condition
