# Supplementary material for: AlertGS: determining alerts for gene sets
Source: Bioinformatics. 2025 Apr 3;41(4):btaf133. doi: 10.1093/bioinformatics/btaf133 (PMC12041417; doi:10.1093/bioinformatics/btaf133)
Supplement: btaf133_Supplementary_Data [file btaf133_supplementary_data.zip › AlertGS_SupplementA.pdf]

A Supporting Information for 'AlertGS: Determining alerts for gene sets': Details on the statistical methods

MCP-Mod Procedure

The MCP-Mod (Multiple Comparison Procedure and Modelling) approach was originally developed for dose finding in Phase II clinical studies [4]. It combines a multiple comparison procedure, i.e. a significance testing step, in which the presence of some relevant signal in the data is established. Only if this is the case, suitable candidate models are fitted to the data, and the best model is determined either via model selection or via model averaging. [5] showed the suitability of this approach also for high-dimensional gene expression data. The methodology and the choice of parameters are explained in the following, for more details, the reader is referred to [4, 2]. An implementation of the methodology is given in the `R` package `DoseFinding` [3].

In this work, a relationship between the time (as predictor variable) and gene expression values (as response variable) is modeled. Thus, in the following the term time-response model is used. Consider k time points $t_1 < t_2 < \ldots < t_k$ with n_i replicates for time point t_i. The model equation for the gene expression value Y_{ij} for time point t_i, $i = 1, \ldots, k$ and replicate $j = 1, \ldots, n_i$ is given by

$$Y_{ij} = \mu_{t_i} + \varepsilon_{ij} \quad \text{with} \quad \varepsilon_{ij} \sim \mathcal{N}(0, \sigma^2),$$

and $\sigma^2 > 0$, independent of the time point. Here it is assumed that $\mu_{t_i} = f(t_i, \boldsymbol{\theta})$ follows a time-response model with parameter vector $\boldsymbol{\theta}$, and that ε_{ij} describes independent errors.

In the first step of the procedure, a multiple comparison procedure (MCP) is performed, where for a set of pre-specified candidate models $\mathcal{M}$, it is tested for each gene individually, whether the profile of the time-response relationships deviates from a flat profile in the direction of any of these candidate models. If this test yields a significant result, following the terminology introduced in [5], a signifMCP (significant result of the MCP) is established for the respective gene. Let $\mathcal{M}$ contain M candidate models. Table A.1 gives the model functions of the fixed set $\mathcal{M}$ in this work. All models can be reformulated as

$$f(t, \boldsymbol{\theta}) = \vartheta_0 + \vartheta_1 f^0(t, \boldsymbol{\theta}^0),$$

Model	$f(t,\boldsymbol{\theta})$	$f^0(t,\boldsymbol{\theta}^0)$	$\boldsymbol{\theta}^0$
linear	$E_0+\delta t$	t	$\emptyset$
exponential	$E_0+E_1\big(\exp(t/a)-1\big)$	$\exp(t/a)-1$	$a=15.38647$
quadratic	$E_0+\beta_1 t+\beta_2 t^2$	$t+(\beta_2/\lvert\beta_1\rvert)t^2$	$\delta=\beta_2/\lvert\beta_1\rvert=-0.02083333$
emax	$E_0+E_{max}t/(ET_{50}+t)$	$t/(ET_{50}+t)$	$ET_{50}=20$
sigEmax	$E_0+E_{max}t^h/(ET_{50}^h+t^n)$	$t^b/(ET_{50}^h+t^h)$	$ET_{50}=24$, $h=5.261537$
Beta	$E_0+E_{max}B(a_1,a_2)\cdot$ $(t/D)^{a_1}(1-t/D)^{a_2}$	$B(a_1,a_2)(t/D)^{a_1}(1-t/D)^{a_2}$	$a_1=2, a_2=1$

Table A.1: Time-response models $f(t,\boldsymbol{\theta})$, their standardized versions $f^0(t,\boldsymbol{\theta}^0)$, and the guesstimates for $\boldsymbol{\theta}^0$ for the analysis. For the beta model, B is defined as $B(a_1,a_2)=(a_1+a_2)^{a_1+a_2}/a_1^{a_1}a_2^{a_2}$ and $D=57.6$.

see the second column of Table A.1. $f^0(t,\boldsymbol{\theta}^0)$ denotes the standardized model function with corresponding parameter vector $\boldsymbol{\theta}^0$, while ϑ_0 denotes a location- and ϑ_1 a scale-parameter.

The shape of the model functions is fully characterized by the parameter vector $\boldsymbol{\theta}^0$. For this parameter vector, guesstimates are determined to provide plausible models used for testing. Here, the same guesstimates need to be used for all genes. Thus, the guesstimates are chosen so that a broad range of possible time-response shapes is covered. Here, for the exponential model, it is assumed that 50% of the overall effect is achieved at time point 38. For the quadratic model, it is assumed that 100% of the effect is achieved at time point 24. For the Emax model, 50% of the overall effect is assumed to be achieved at time point 20, and for the sigEmax model, 50% and 95% of the effect are assumed to be achieved at time points 24 and 42, respectively. A graphical representation of the resulting candidate profiles is given in Figure A.1.

To test for a signifMCP, for each gene and each candidate model, a contrast test is performed. For $m=1,\dots M$ indicating the m-th model with standardized model $f_m^0(t,\boldsymbol{\theta}^0)$, the hypothesis $H_m^0:\boldsymbol{c_m^T}\boldsymbol{\mu}_m=0$ versus the alternative $H_m^1:\boldsymbol{c_m^T}\boldsymbol{\mu}_m\neq 0$ is tested. $\boldsymbol{c_m}=(c_{m1},\dots c_{mk})^T$ denotes the optimal contrast vector of the m-th model, and $\boldsymbol{\mu}_m=(\mu_{m1},\dots,\mu_{mk})^T$ denotes the mean response vector based on the standardized model f_m^0. The optimal contrast vector is chosen in a way to maximize the power of the test for the assumed model shape [2].

The test statistics are given by

$$T_m=\frac{\sum_{i=1}^k c_{mi}\bar{Y}_i}{S\sqrt{\sum_{i=1}^k \frac{c_{mi}^2}{n_i}}},\quad m=1,\dots,M,$$

with $\bar{Y}_i$ denoting the mean at time point t_i and $S^2=\sum_{i=1}^k\sum_{j=1}^{n_i}(Y_{ij}-\bar{Y}_i)^2/(N-k)$ denoting the pooled variance, where $N=\sum_{i=1}^k n_i$. Unter the null hypothesis, the vector of test statistics, $(T_1,\dots,T_M)^T$ follows a central multivariate t-distribution. For $q_{1-\alpha}$ denoting the equicoordinate

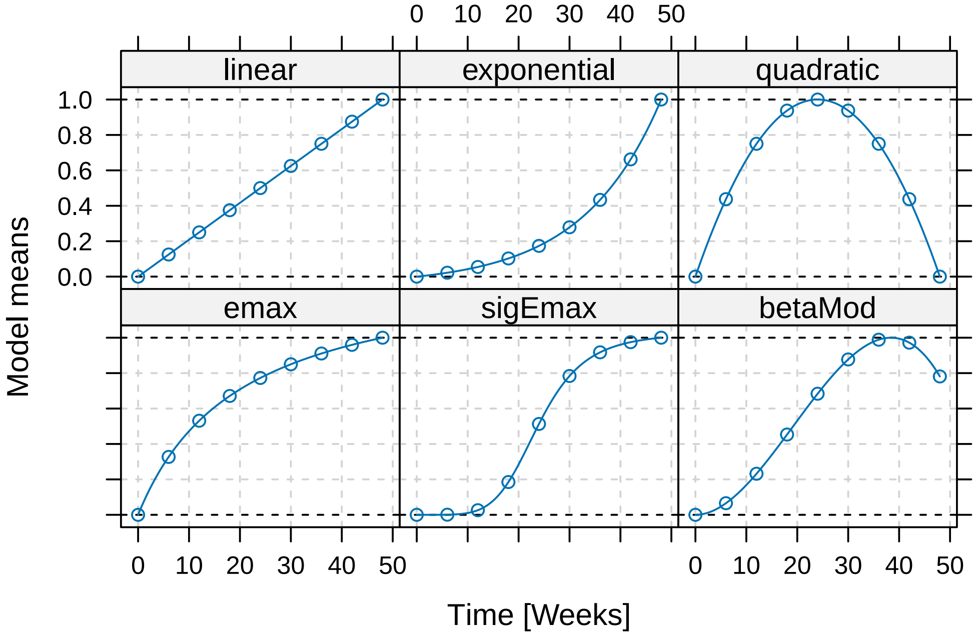

Figure A.1: Graphical display of the assumed model shapes for the MCP step in the MCP-Mod methodology.

(1-α)-quantile of this distribution, a signifMCP is established if $T_{\max} = \max(T_1, \ldots, T_M) > q_{1-\alpha}$. All models with $T_m > q_{1-\alpha}$ are considered to be statistically significant in this multiple testing context, while controlling the family-wise error rate. These models form the set $\mathcal{M}^* \subset \mathcal{M}$ of L significant models.

Only if for at least one model, a signifMCP is established, the time-response relationship for the respective gene is modelled. Here, the parameters for all models in $\mathcal{M}^*$ are estimated using a numeric optimization algorithm. The final 'winner model' is selected based on the AIC values of all fitted models.

In the implementation of the MCP-Mod methodology in the `DoseFinding` package in `R`, for the calculation of the MCP, the lowest condition is required to have a value of 0. In the data considered in this work, however, the lowest time point is at 3 weeks. Thus, for the calculation of candidate models and power-optimal contrasts, this value is set to 0. Only the calculation of the signifMCP is affected by this via the adjustment of the optimal contrasts. The modelling step including the AIC-based model selection is then again performed on the original data, which particularly means that the final fitted model and the calculation of the ALEC is based on the un-modified data.

The quality of the model fit can be assessed via the adjusted coefficient of determination R^2_{adj}, which for a model fit $f(t, \hat{\boldsymbol{\theta}})$ and response data y_{ij}, $i = 1, \ldots, k$, $j = 1, \ldots, n_i$ for a specific gene is defined as

$$R^2_{\text{adj}} = 1 - \frac{(1 - R^2)(N - 1)}{N - p}, \quad R^2 = 1 - \frac{\sum_{i=1}^{k} \sum_{j=1}^{n_i} (y_{ij} - f(t_i, \hat{\boldsymbol{\theta}}))^2}{\sum_{i=1}^{k} \sum_{j=1}^{n_i} (y_{ij} - \bar{y})^2},$$

with p denoting the number of parameters.

Graphical Summary of the AlertGS Methodology

The entire AlertGS methodology is based on gene-wise alerts, obtained via some modeling approach. Then, the steps alert preparation, gene set annotation, test statistic calculation, global p-value determination, and AlertGS calculation follow. If applicable for the respective type of gene sets considered, e.g. for GO groups, a decorrelation approach is used to handle the high overlaps between gene sets. In the following, the procedure is visualized based on the data from the case study presented in the main manuscript, for one chosen GO group.

The GO group chosen for this example has the ID GO:0019233, with GO term ‘sensory perception of pain’. There are 97 genes annotated in this group, 22 of which have an alert.

The alert preparation contains the imputation of missing values, the replacing of alerts in the ‘wrong direction’ (i.e. alerts for a down-regulation instead of an up-regulation), the breaking of ties, and the sorting in increasing order. Genes without an observed alert, or with an observed alert in the wrong direction, are assigned alerts outside the range of observable values. Specifically, for genes without an alert, an imputation is obtained by randomly sampling from the interval $[c_{\max} + \varepsilon, 2 \cdot c_{\max} - \varepsilon]$, for $\varepsilon > 0$ and $c_{\max}$ denoting the maximal tested condition value, which in this case corresponds to 48. Genes with an alert in the ‘wrong direction’ obtain an imputed value of $3 \cdot c_{\max} -$alert. This approach is visualized in Figure A.2. Ties are broken by adding a very small random number to duplicate values.

A graphical display of the resulting gene-wise alerts is given in Figure A.3, for the genes not annotated to the considered GO group (top), and the genes annotated to the considered group (bottom). The red vertical lines indicate the three segments of true alerts (first segment, up to week 48), with imputed missing alerts (second segment, week 48 to week 96) and with imputations for alerts in the wrong direction (third segment, starting at week 96). Overall, the

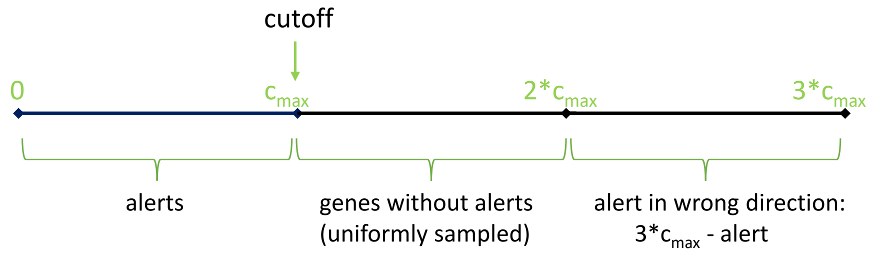

Figure A.2: Graphical visualization of the imputations for the preparation of the gene-wise alerts.

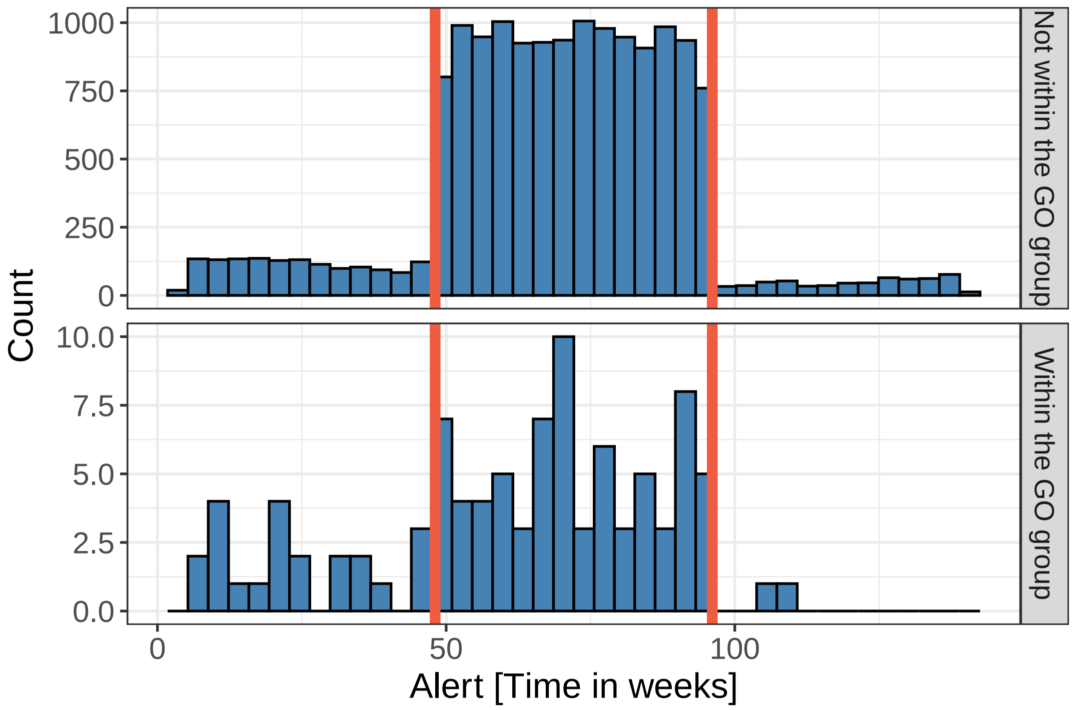

Figure A.3: Distribution of the alerts for all genes within the considered GO group (bottom) and all other genes not within the considered GO group (top). Note the different y-axes of the plots. The red vertical lines indicate the time points 48 weeks and 96 weeks, such that the first segment (up to week 48) shows the actual alerts, the second segment (week 48 to 96) shows the imputed values for missing alerts, and the third segment (from week 96) shows the transformed alerts in the wrong direction.

higher number of genes with missing alerts in comparison to the observed alerts in either direction becomes obvious.

In the second step of the overall procedure, the gene set annotation is performed. This is not visualized in this example, but for this step, all gene sets of interest (GO groups in the example) and their respective annotated genes are defined.

In the third step, the running sum test statistic is calculated for each considered group. The resulting trajectory of the running sum test statistic is shown in Figure A.4. The blue vertical line indicates the threshold of 48 weeks, until which the maximum value of the test statistic is evaluated. The corresponding maximum value of the test statistic is indicated by a red dot. The value of the test statistic is 167.4, and it is attained for the time (i.e. the alert) 44.62.

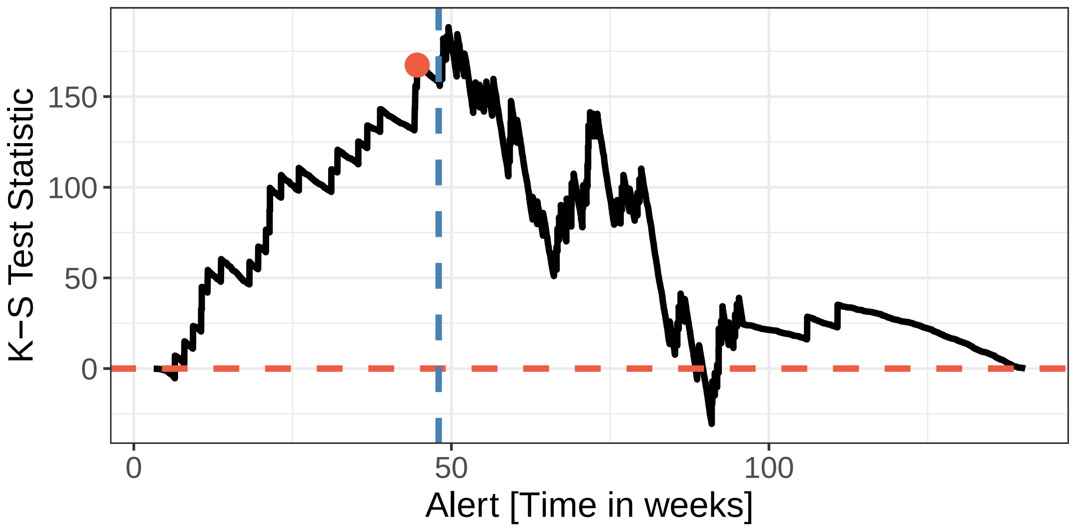

Figure A.4: Result of the running sum test statistic for the considered GO group, together with the indicated maximum value (red dot) before the threshold of 48 weeks (blue vertical line).

For the calculation of the global p-value, the label of whether a gene (corresponding to a certain alert) is within the considered GO group is randomly permuted 1000 times. For each permutation, the running sum test statistic is calculated and its respective maximal value is determined. A graphical example of the first 100 permutation runs superimposed to the original Kolmogoriv-Smirnoff test statistic is shown in Figure A.5. In addition to the test statistic values, also the respective maximum value of the test statistic is indicated by a transparent red dot.

These maximum values for all 1000 iterations are shown in the histogram in Figure A.6. Additionally, the density of the estimated rectified Gumbel distribution is shown in the plot, together with a vertical line indicating the value of the observed maximum value of the test statistic. The resulting p-value, based on the fitted Gumbel distribution, is 0.0005426885, yielding a global significant result. This means that the GO group contains more genes with alerts than expected at random.

After determining the global significance of the group, the AlertGS is determined. This is based directly on the trajectories of the permutation-based Kolmogorov-Smirnoff test statistics. Figure A.7 shows the relevant range of the Kolmogorov-Smirnoff test statistic, together with the result of the first 100 permutations. The four indicated points P1, P2, P3, P4 correspond to the first four alert times of genes within the GO group. These are the alerts, where the respective p-values of the permutations are calculated.

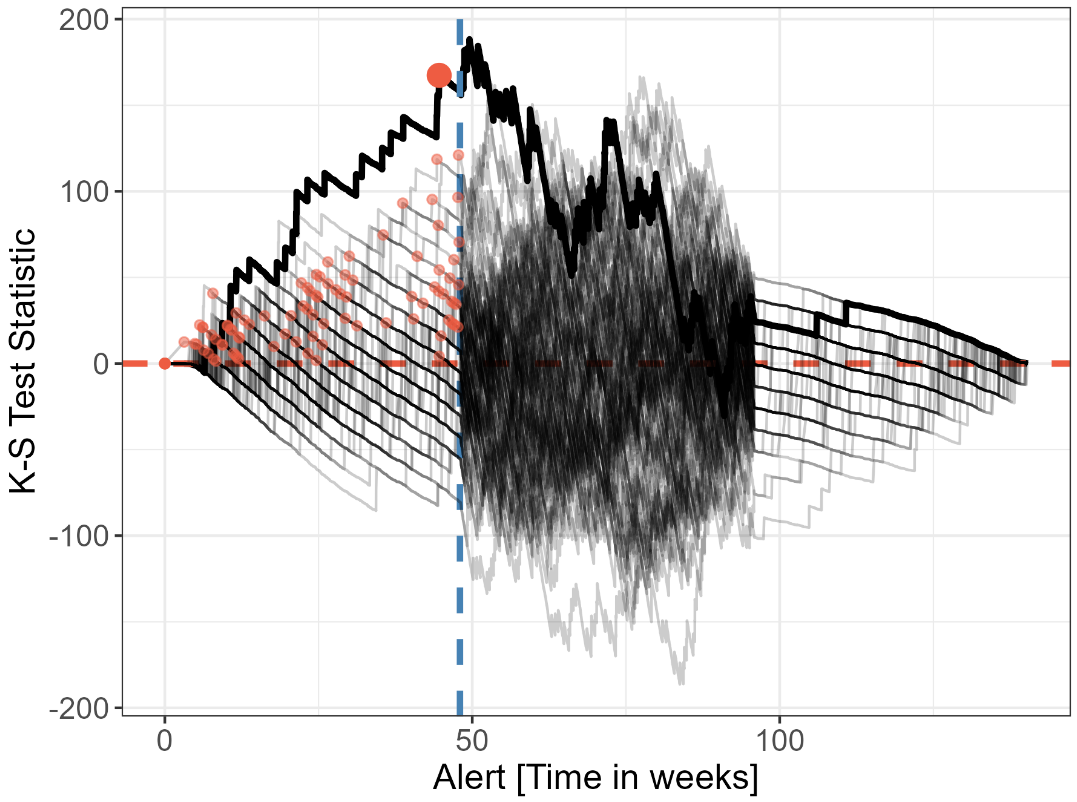

Figure A.5: Result of the running sum test statistic as shown in Figure A.4 together with the indicated permutation-based running sum test statistics for the first 100 permutation runs. Maximal values of the permutation-based test statistics are indicated by transparent red dots.

At the alerts P1 through P4, the value of the running sum test statistic on the real data is compared to the values of the 1000 running sum test statistics based on the permuted data. Figure A.8 shows the respective histograms of the observed permutation-based running sum test statistics at the alerts P1, P2, P3 and P4. The respective p-value is calculated as the ratio of permutation-based test statistics larger than or equal to the test statistic value on the real data. This value is compared to the significance level of 5%. Here, point P4 is the first point where the p-value is smaller than 5%, so the AlertGS for the entire group is given by the time corresponding to P4, which is 10.61.

To determine whether the group is also the local minimum, all children and parent nodes of the considered group are assessed. The children are the groups with identifiers GO:0051930 and GO:0062149, and the only parent is the group GO:0007600. Their global p-values are 0.0036, 0.0387, and 0.5445, respectively. In comparison to the global p-value of the GO group under consideration, which was 0.0005, these are all larger, such that the considered GO group also fulfills this local minimum condition. The respective children, even though both of them

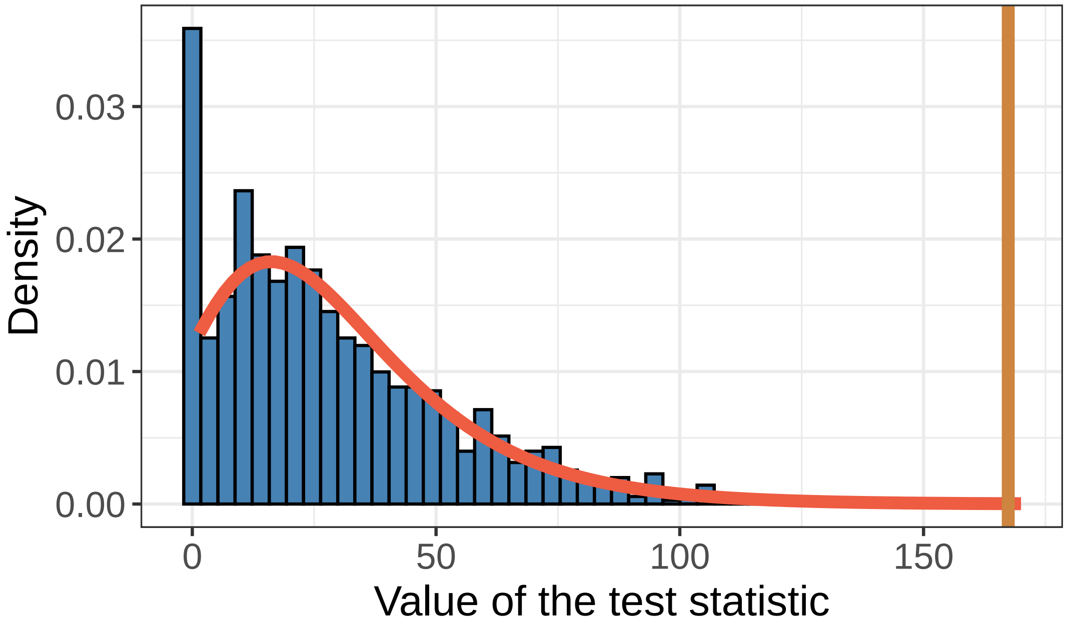

Figure A.6: Histogram of the resulting maximum values of the permutation test statistics, together with the fitted (rectified) Gumbel distribution. The observed value of the test statistic for the real data is indicated by the brown vertical line.

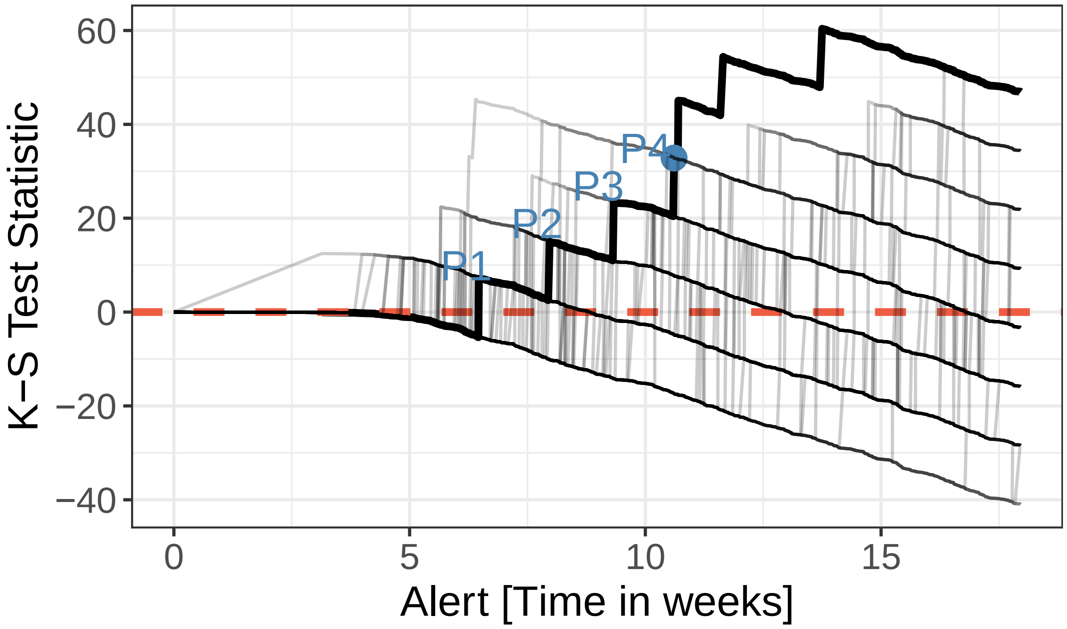

Figure A.7: Relevant range of the entire Kolmogorov-Smirnoff test statistic with superimposed trajectories of the first 100 permutations for the calculation of the AlertGS. The first four alerts are indicated by points P1 to P4. The final AlertGS at P4 is indicated by a blue dot.

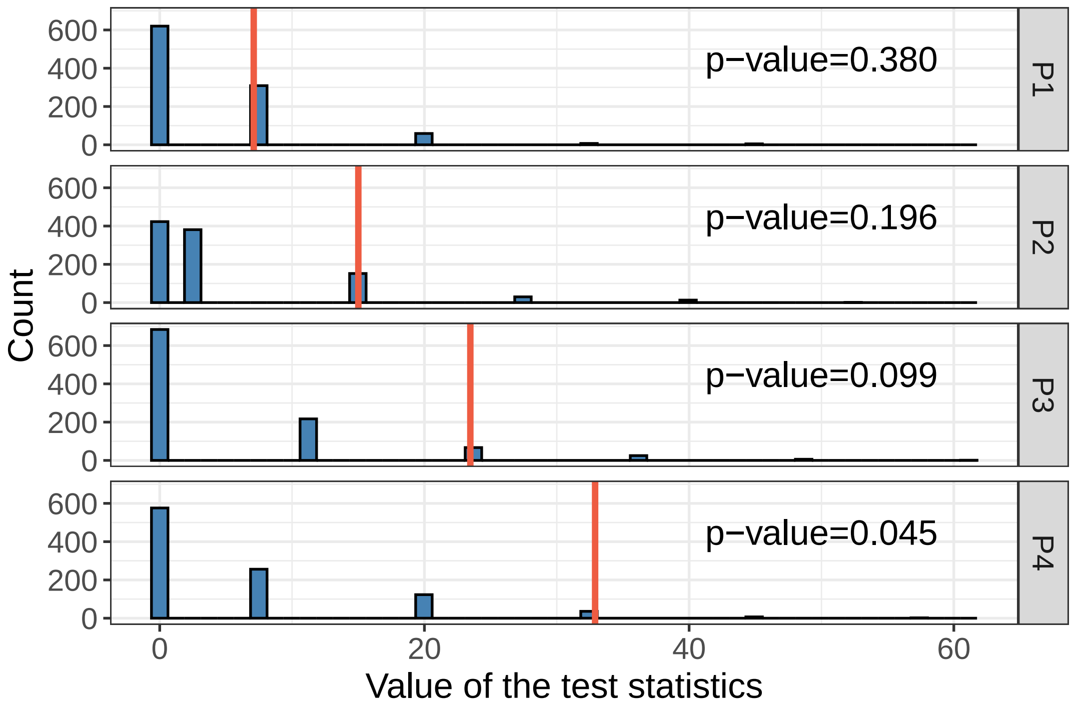

Figure A.8: Histograms of the observed values of the permutation-based test statistics at the first four alerts of genes within the GO group. The red vertical line indicates the test statistic of the real data at the respective points.

have a global p-value smaller than 5%, are correspondingly not considered to be significant in the sense of the local minimum, since at least one of their parents, namely the considered GO group, has a lower p-value.

Fisher's Test-based GO Analysis

The results of the controlled simulation study, obtained by applying the AlertGS methodology, are compared to the results when applying Fisher's test. The general approach of Fisher's test-based GO analysis, tailored to the specific situation at hand, is as follows [6]. For all genes, some threshold for each gene is defined. In the case here, the threshold is based on the gene-wise alert, and the specific threshold value is set to 20. In the general application, the threshold is often based on the p-value for testing for differential over- or underexpression. For each gene set of interest, a 2x2 contingency table is created, stating the number of genes within the gene set with alert values below and above the threshold, and the number of genes not in the gene set with alert values below and above the threshold. To test for under- or overrepresentation of the genes below the threshold in the given gene set, Fisher's exact test is conducted. Significant results for this test indicate that more/fewer genes within the gene set are below the threshold than expected at random.

Specifically in the case where the considered gene sets are defined by gene ontology (GO) groups, high correlations between the groups due to the hierarchical structures are present. Thus, [1] proposed several approaches for decorrelating the results of such GO analyses. In this work, the elim (short for elimination) approach is used. This approach works in a way, that all GO groups are assessed bottom-up, i.e. starting with the most specialized groups. If a GO group yields a significant result of the Fisher test, then all genes annotated to this group are removed from all ancestor nodes of the significant group.

References

[1] A. Alexa, J. Rahnenführer, and T. Lengauer. Improved scoring of functional groups from gene expression data by decorrelating GO graph structure. *Bioinform.*, 22(13), 2006.

[2] B. Bornkamp, J. Pinheiro, and F. Bretz. MCPMod: An R Package for the Design and Analysis of Dose-Finding Studies. *Journal of Statistical Software*, 29(7), 2009.

[3] B. Bornkamp et al. *DoseFinding: Planning and Analyzing Dose Finding Experiments*, 2023. R package version 1.1-1.

[4] F. Bretz, J.C. Pinheiro, and M. Branson. Combining Multiple Comparisons and Modeling Techniques in Dose-Response Studies. *Biom.*, 61(3), 2005.

[5] J.C. Duda, F. Kappenberg, and J. Rahnenführer. Model selection characteristics when using MCP-Mod for dose–response gene expression data. *Biom. J.*, 64(5), 2022.

[6] P. Khatri, M. Sirota, and A.J. Butte. Ten years of pathway analysis: current approaches and outstanding challenges. *PLOS Comput. Biol.*, 8(2), 2012.
