# Supplementary material for: AlertGS: determining alerts for gene sets
Source: Bioinformatics. 2025 Apr 3;41(4):btaf133. doi: 10.1093/bioinformatics/btaf133 (PMC12041417; doi:10.1093/bioinformatics/btaf133)
Supplement: btaf133_Supplementary_Data [file btaf133_supplementary_data.zip › AlertGS_SupplementC.pdf]

C Supporting Information for 'AlertGS: Determining alerts for gene sets': Additional Figures and Tables

Additional Results for the Descriptive Analysis of the Case Study

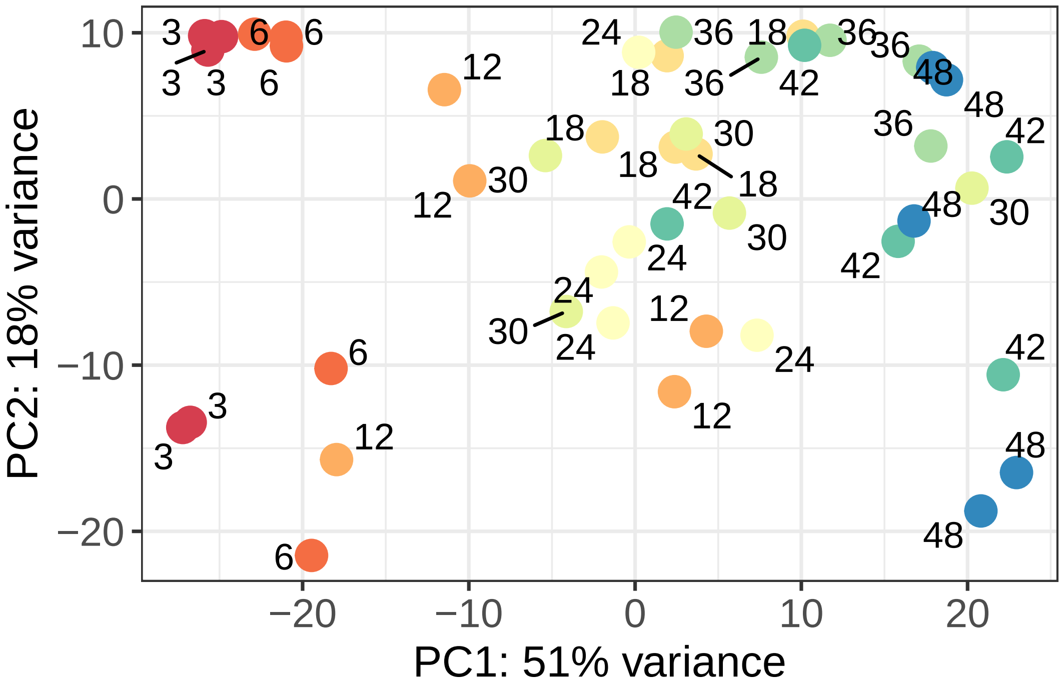

Figure C.1: Graphical display of the first two principal components for the 500 genes with the highest variability across all samples, after performing a variance stabilizing transformation. The respective weeks are indicated by colors and corresponding labels.

Additional Results for the Simulation Study

The additional results for the simulation study shown here summarize the main analyses also conducted in the main manuscript.

Here, the simulation scenario $x = 0.5$ is shown additionally for the iterative approach, and all scenarios for the independent approach are shown. Further, all results are also shown for the unadjusted p-values, i.e. without applying the LocMin procedure. The structure mainly follows the structure of the main manuscript.

Number of false positive GO groups in the null situation

The following plot corresponds to Figure 3 from the main manuscript but shows the situation without the LocMin adjustment.

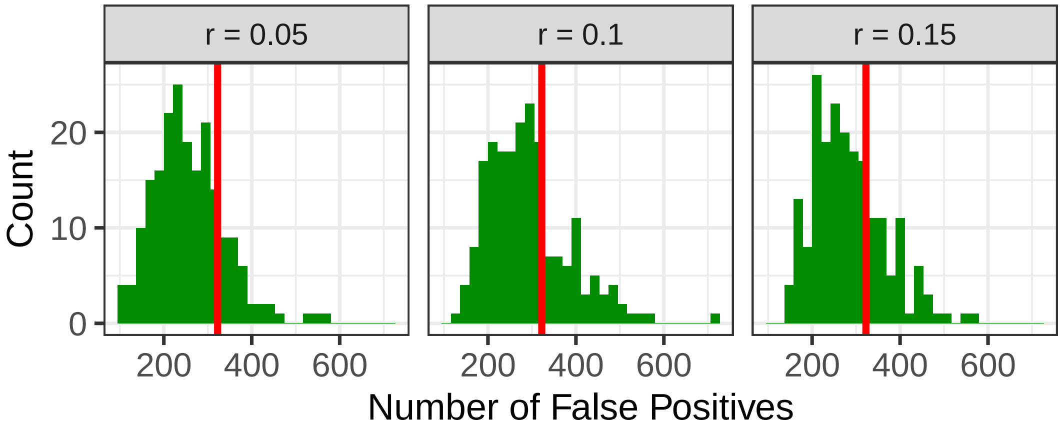

Figure C.2: Number of false positive GO groups in the null situation, without adjustment, with three different percentages of genes with uniformly sampled alerts. The vertical line indicates the level of 5%

Summary of the true positives and the overall number of groups with significant result

The following plots correspond to Figure 4 from the main manuscript.

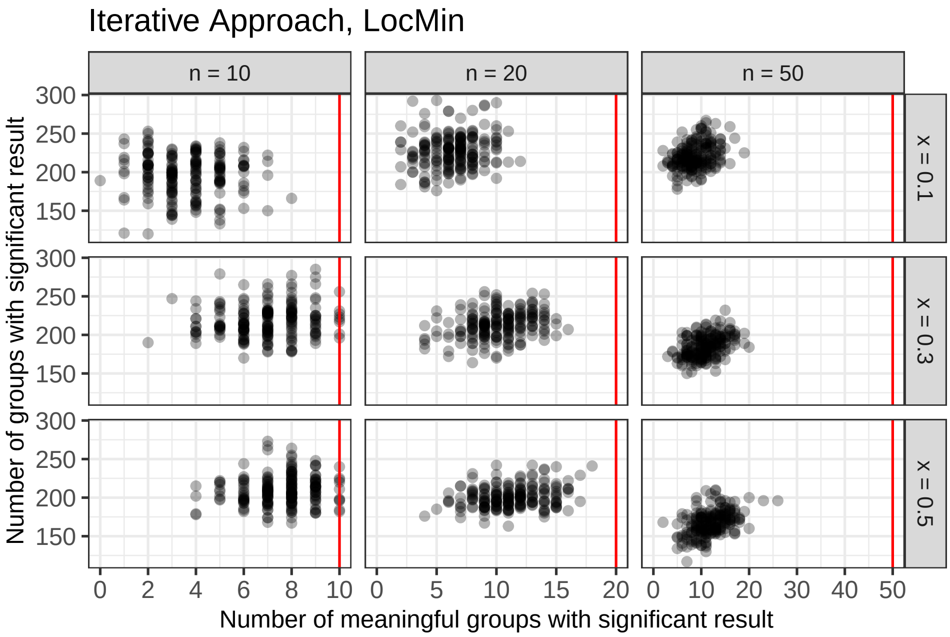

Figure C.3: Summary of the number of true positives, i.e. numbers of meaningful groups with significant results, and the overall number of groups with a significant result, for all simulation scenarios, for the iterative approach with LocMin adjustment. The number of true positives is bounded by the number of meaningful groups in the respective scenario, this is indicated by the vertical red line.

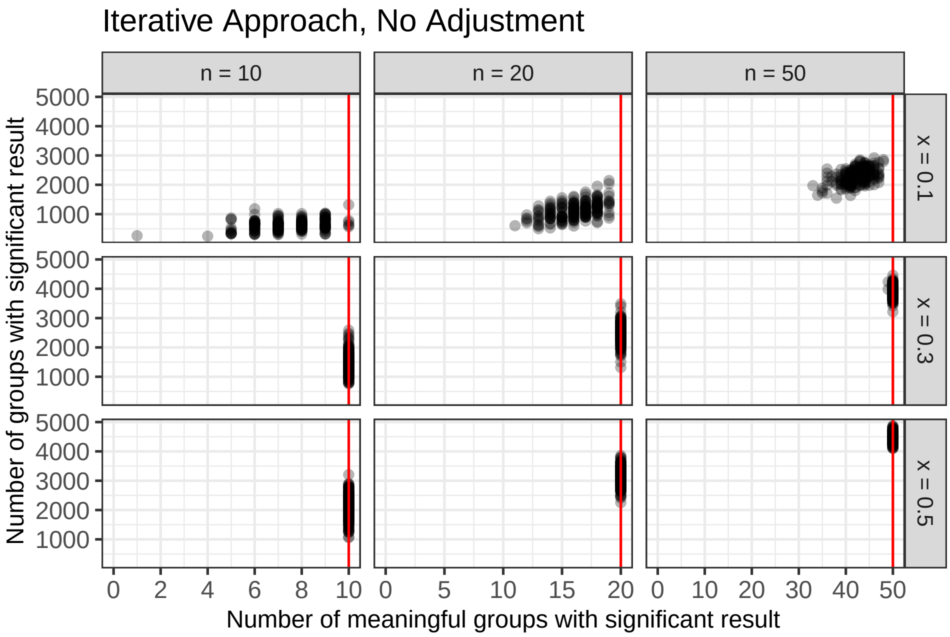

Figure C.4: Summary of the number of true positives, i.e. numbers of meaningful groups with significant results, and the overall number of groups with a significant result, for all simulation scenarios, for the iterative approach without adjustment. The number of true positives is bounded by the number of meaningful groups in the respective scenario, this is indicated by the vertical red line.

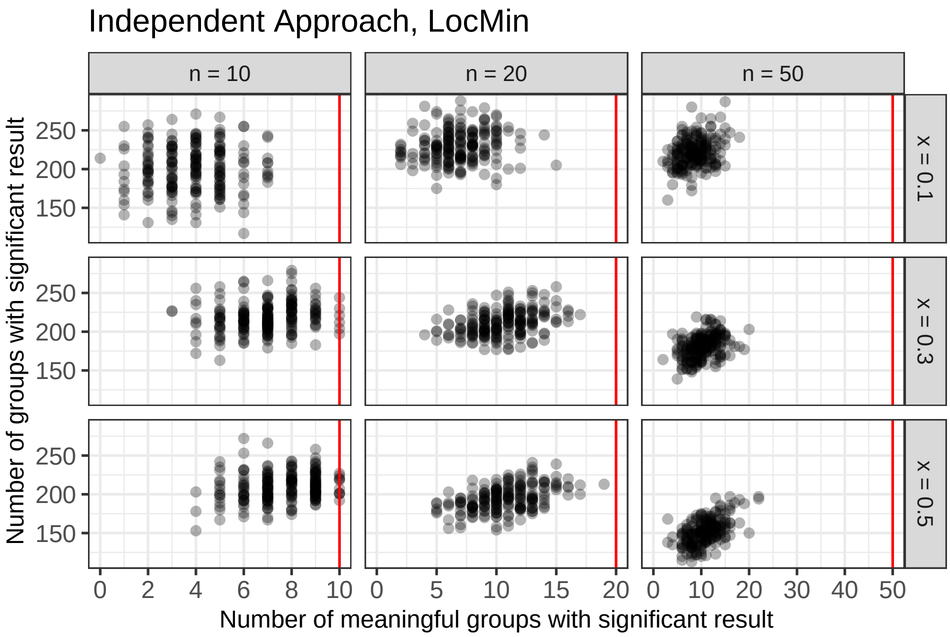

Figure C.5: Summary of the number of true positives, i.e. numbers of meaningful groups with significant results, and the overall number of groups with a significant result, for all simulation scenarios, for the independent approach with LocMin adjustment. The number of true positives is bounded by the number of meaningful groups in the respective scenario, this is indicated by the vertical red line.

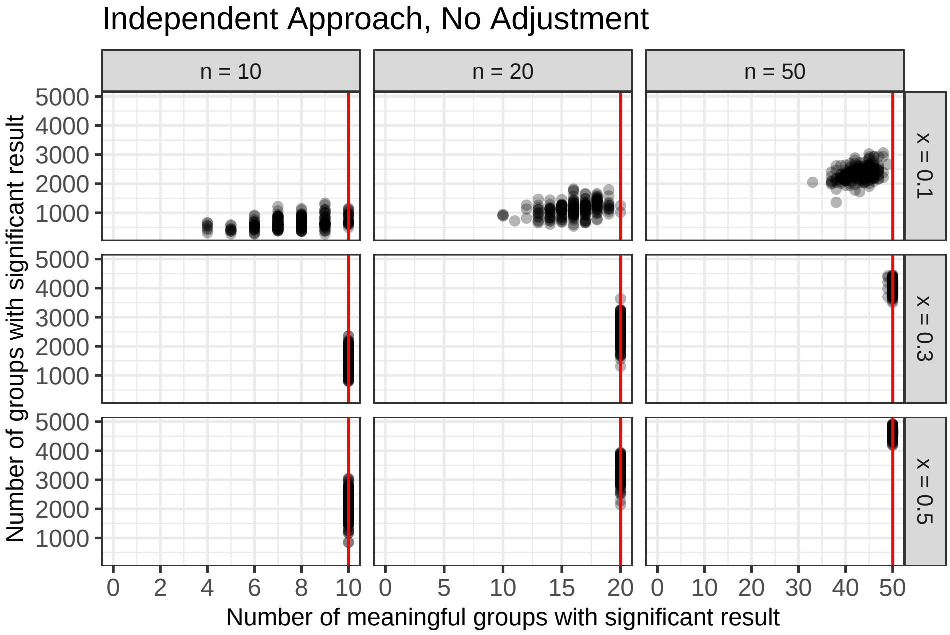

Figure C.6: Summary of the number of true positives, i.e. numbers of meaningful groups with significant results, and the overall number of groups with a significant result, for all simulation scenarios, for the independent approach without adjustment. The number of true positives is bounded by the number of meaningful groups in the respective scenario, this is indicated by the vertical red line.

Number of the top-k identified groups that are meaningful groups or the parent/child of a true meaningful group.

The following plots correspond to Figure 5 from the main manuscript.

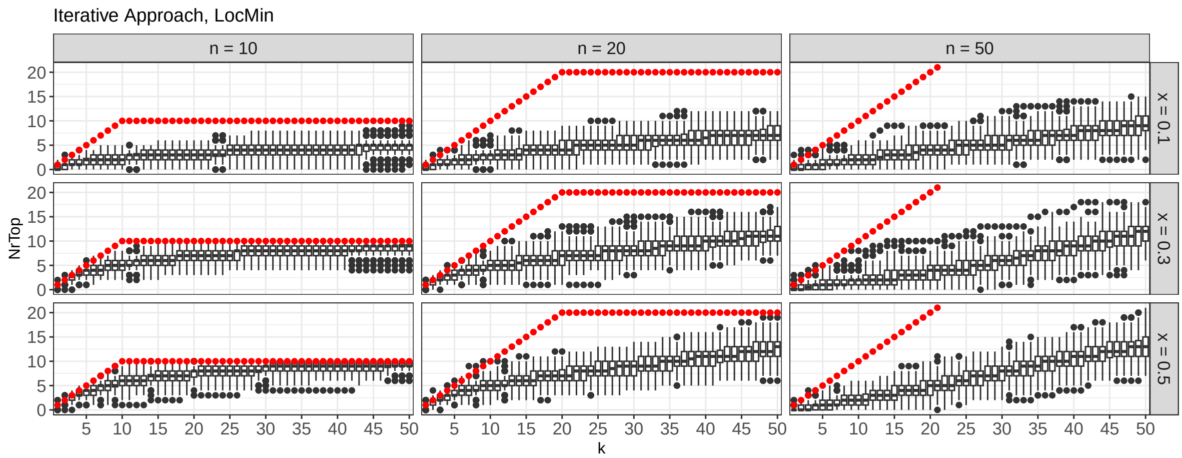

Figure C.7: Boxplots showing for $k = 1, ..., 50$ how many of the top-k identified groups are meaningful groups or the parent/child of a true meaningful group for all scenarios in the iterative approach with LocMin adjustment. The true number of meaningful groups is indicated by red dots. Observed numbers larger than the respective k might occur due to the additional consideration of the parents and children of the true groups.

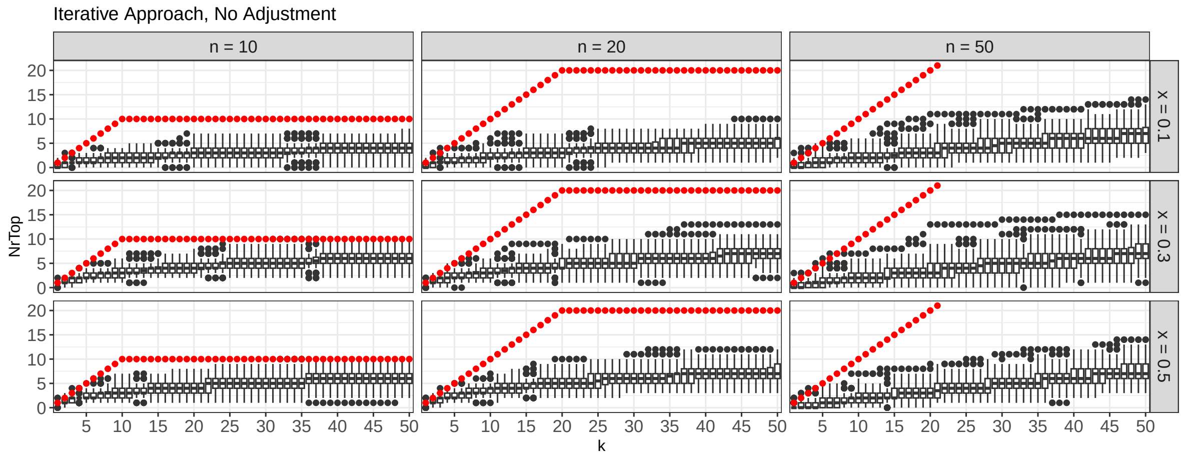

Figure C.8: Boxplots showing for $k = 1, ..., 50$ how many of the top-k identified groups are meaningful groups or the parent/child of a true meaningful group for all scenarios in the iterative approach without adjustment. The true number of meaningful groups is indicated by red dots. Observed numbers larger than the respective k might occur due to the additional consideration of the parents and children of the true groups.

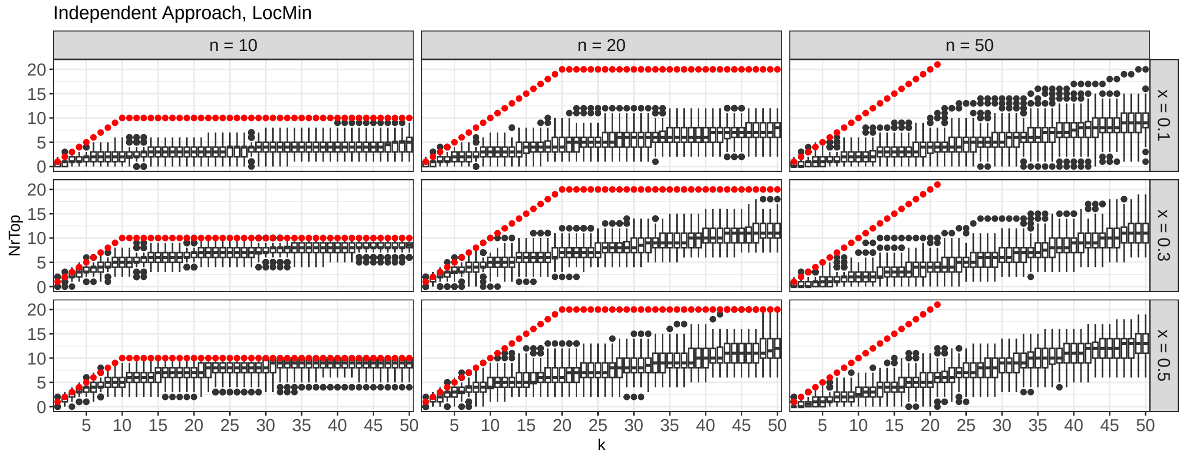

Figure C.9: Boxplots showing for $k = 1, ..., 50$ how many of the top-k identified groups are meaningful groups or the parent/child of a true meaningful group for all scenarios in the independent approach with LocMin adjustment. The true number of meaningful groups is indicated by red dots. Observed numbers larger than the respective k might occur due to the additional consideration of the parents and children of the true groups.

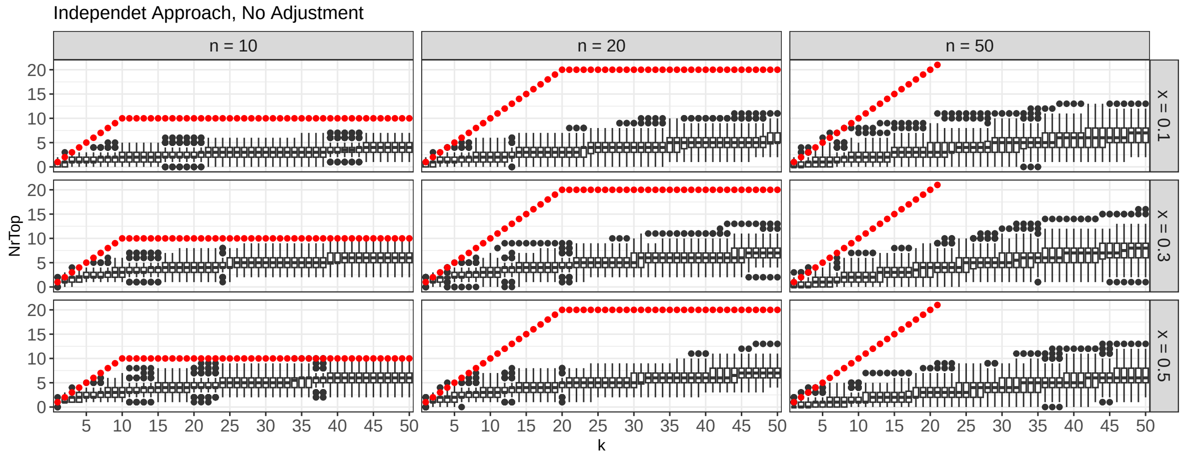

Figure C.10: Boxplots showing for $k = 1, ..., 50$ how many of the top-k identified groups are meaningful groups or the parent/child of a true meaningful group for all scenarios in the independent approach without adjustment. The true number of meaningful groups is indicated by red dots. Observed numbers larger than the respective k might occur due to the additional consideration of the parents and children of the true groups.

Comparison of the estimated AlertGS values and the respective true groupwise alerts

The following plots correspond to Figure 6 from the main manuscript.

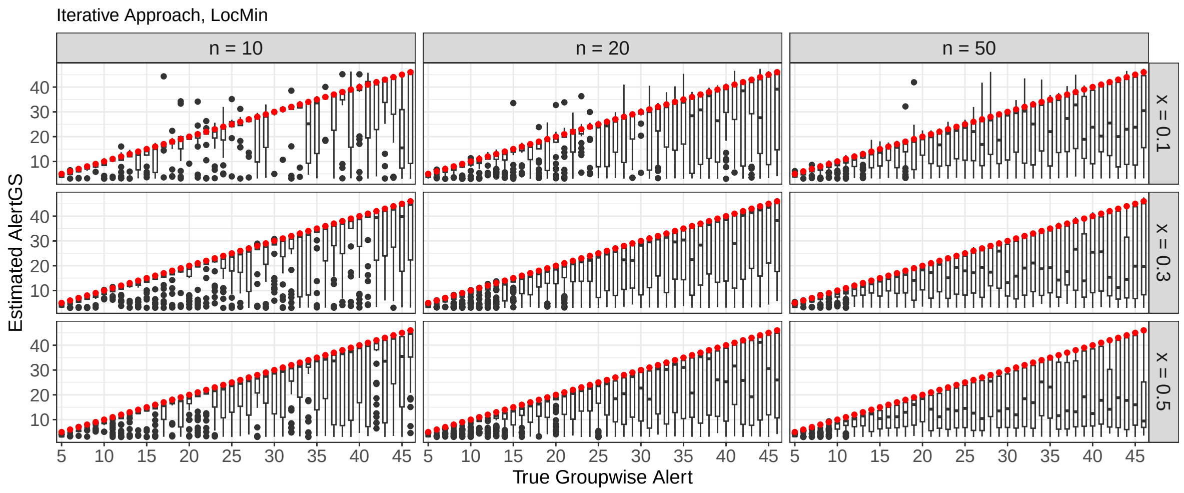

Figure C.11: Comparison of the estimated AlertGS values and the respective true groupwise alerts (red dots) for the correctly identified significant groups for all scenarios and the iterative approach with LocMin adjustment.

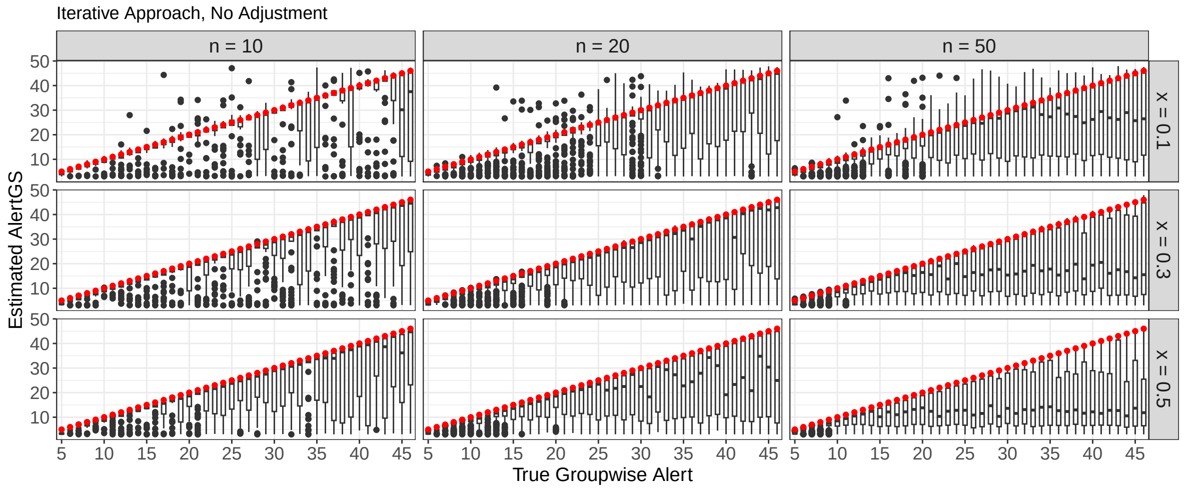

Figure C.12: Comparison of the estimated AlertGS values and the respective true groupwise alerts (red dots) for the correctly identified significant groups for all scenarios and the iterative approach without adjustment.

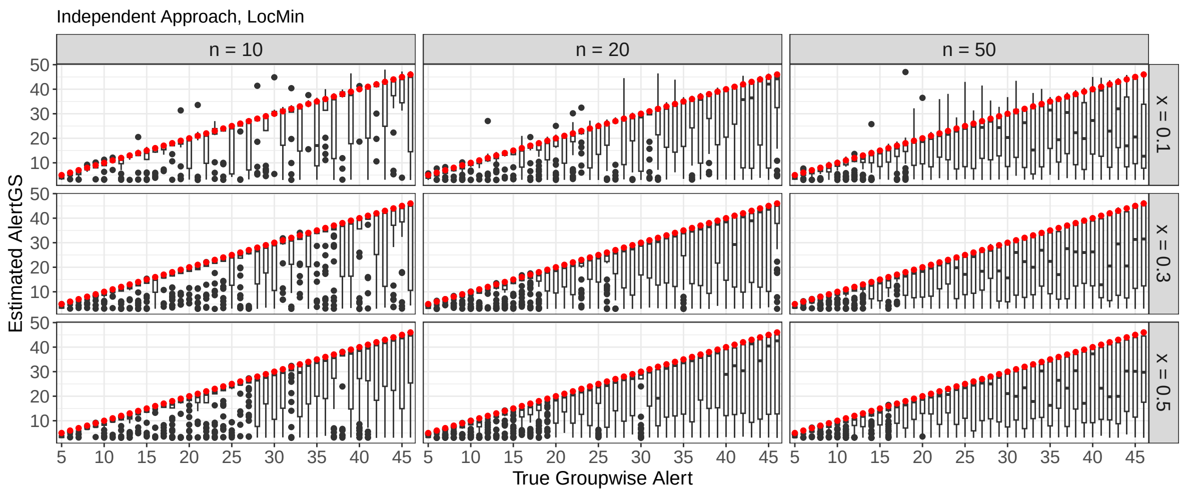

Figure C.13: Comparison of the estimated AlertGS values and the respective true groupwise alerts (red dots) for the correctly identified significant groups for all scenarios and the independent approach with LocMin adjustment.

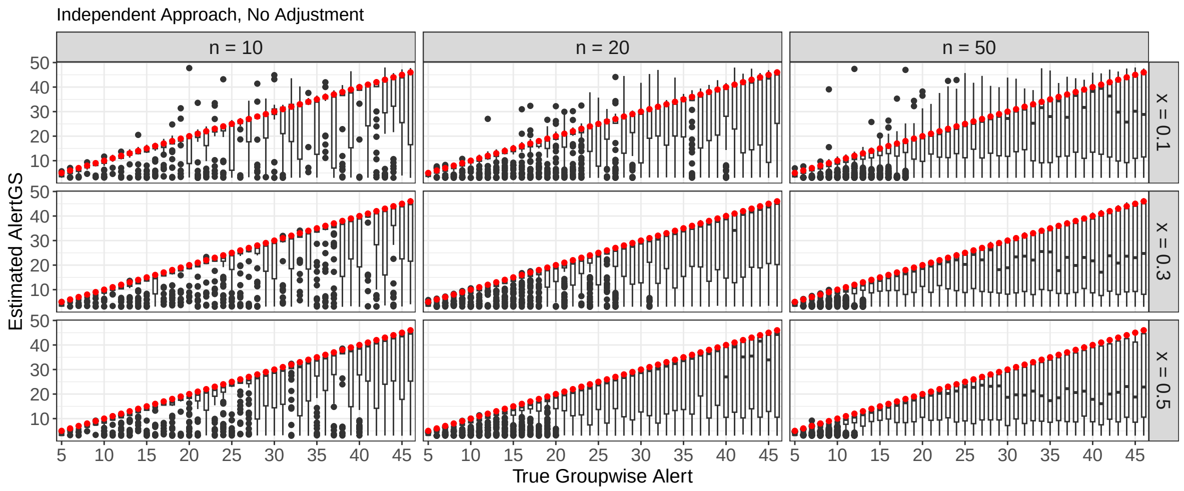

Figure C.14: Comparison of the estimated AlertGS values and the respective true groupwise alerts (red dots) for the correctly identified significant groups for all scenarios and the independent approach without adjustment.

Comparison AlertGS and Elim approach

The following plots correspond to Figure 7 from the main manuscript.

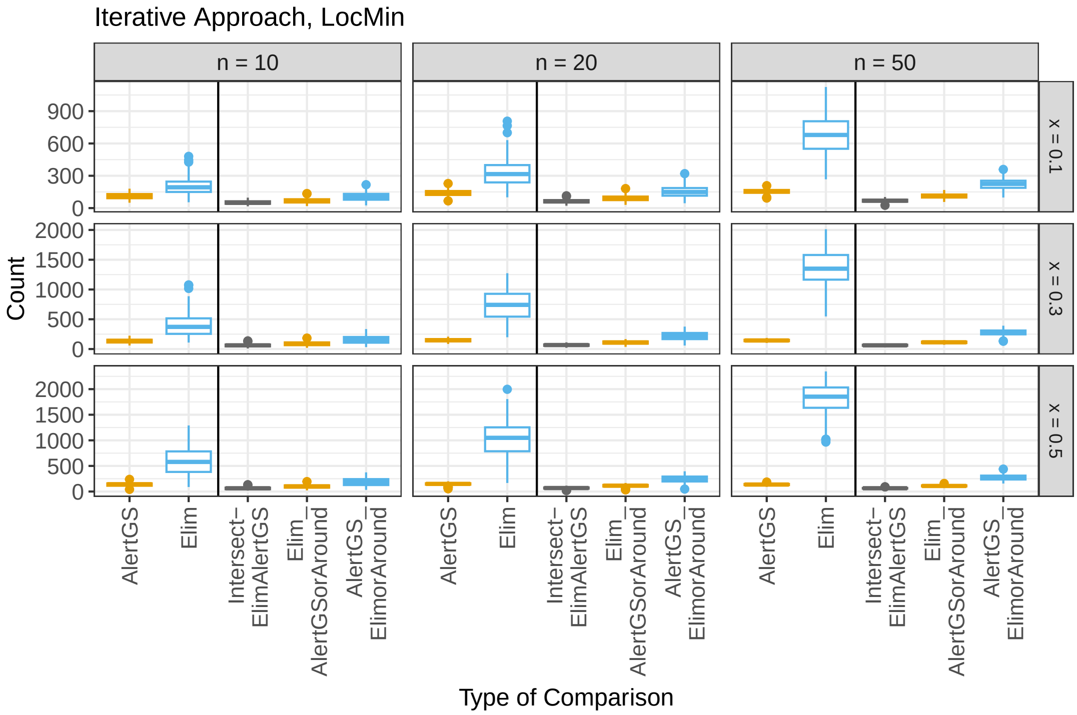

Figure C.15: Boxplots indicating the numbers of identified groups with the AlertGS (iterative approach, LocMin adjustment) and the Elim approach, as well as the number of intersections between these lists, the number of groups found by the AlertGS approach, where either the group itself or a parent/child of the group is also found by the Elim approach, and vice versa.

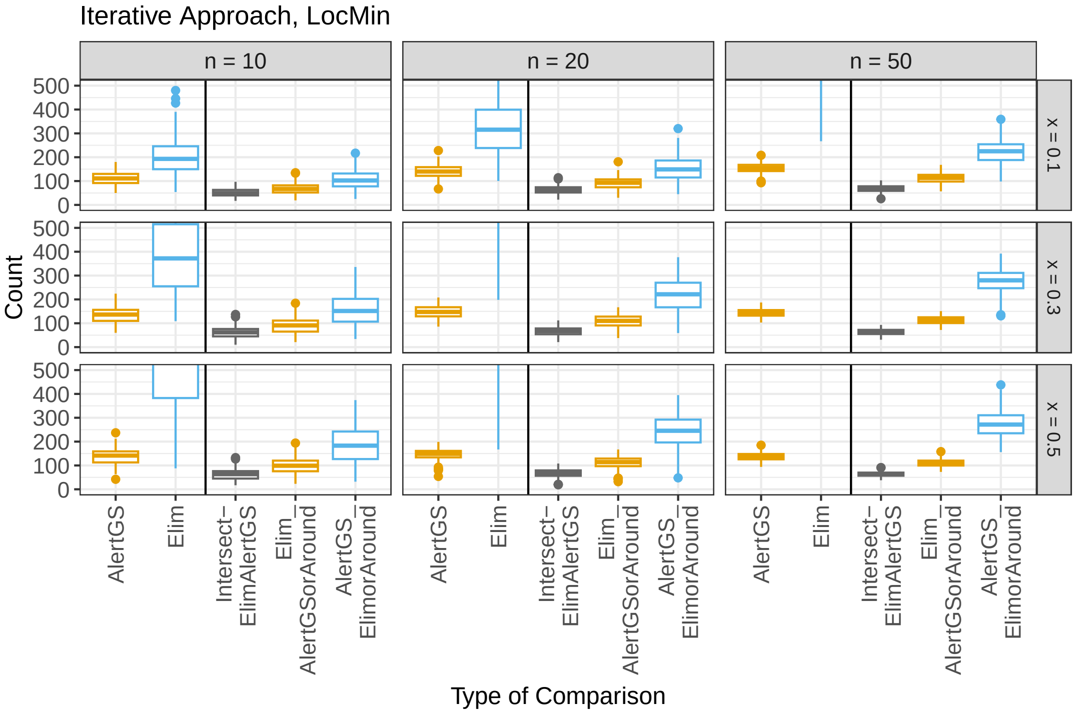

Figure C.16: The same results as shown in Figure C.15, but with a smaller y-axis limit.

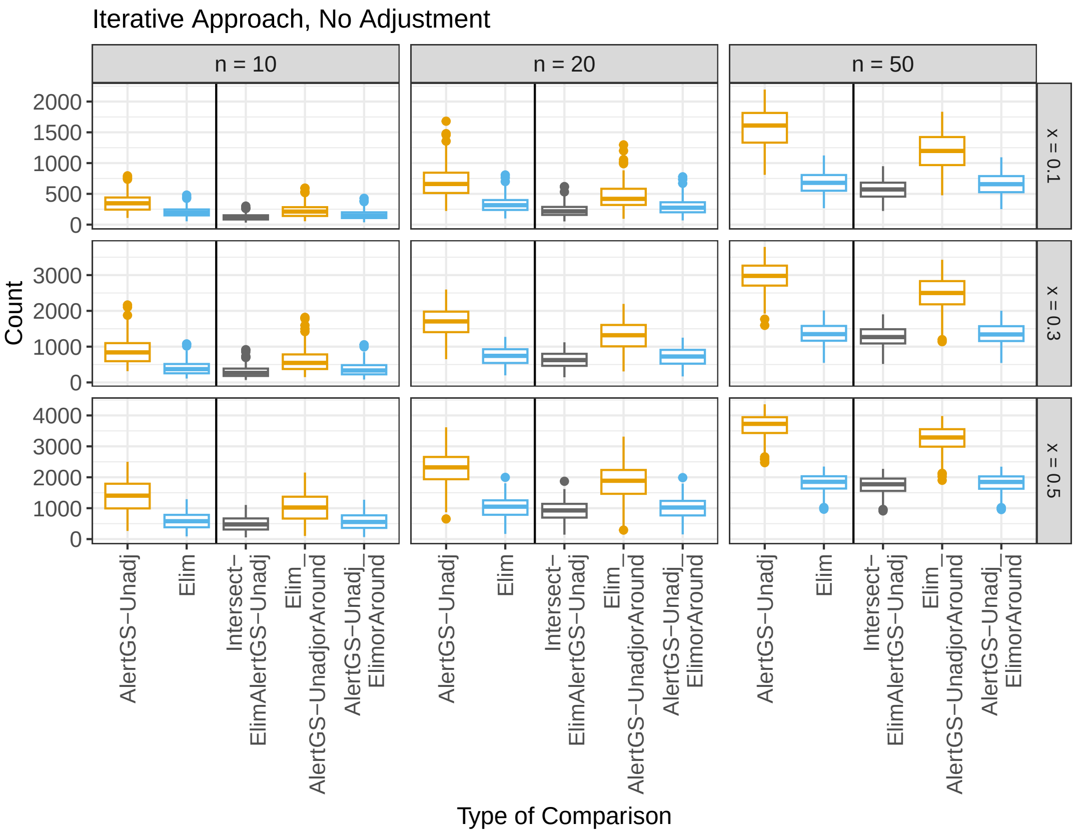

Figure C.17: Boxplots indicating the numbers of identified groups with the AlertGS (iterative approach, without adjustment) and the Elim approach, as well as the number of intersections between these lists, the number of groups found by the AlertGS approach, where either the group itself or a parent/child of the group is also found by the Elim approach, and vice versa.

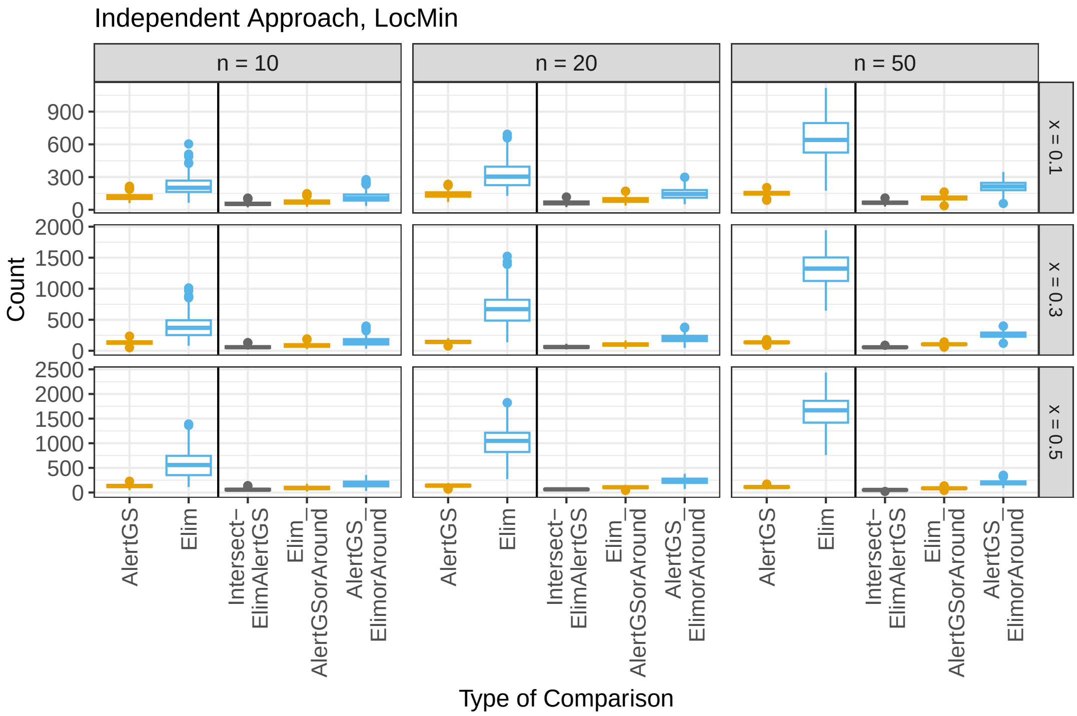

Figure C.18: Boxplots indicating the numbers of identified groups with the AlertGS (independent approach, LocMin adjustment) and the Elim approach, as well as the number of intersections between these lists, the number of groups found by the AlertGS approach, where either the group itself or a parent/child of the group is also found by the Elim approach, and vice versa.

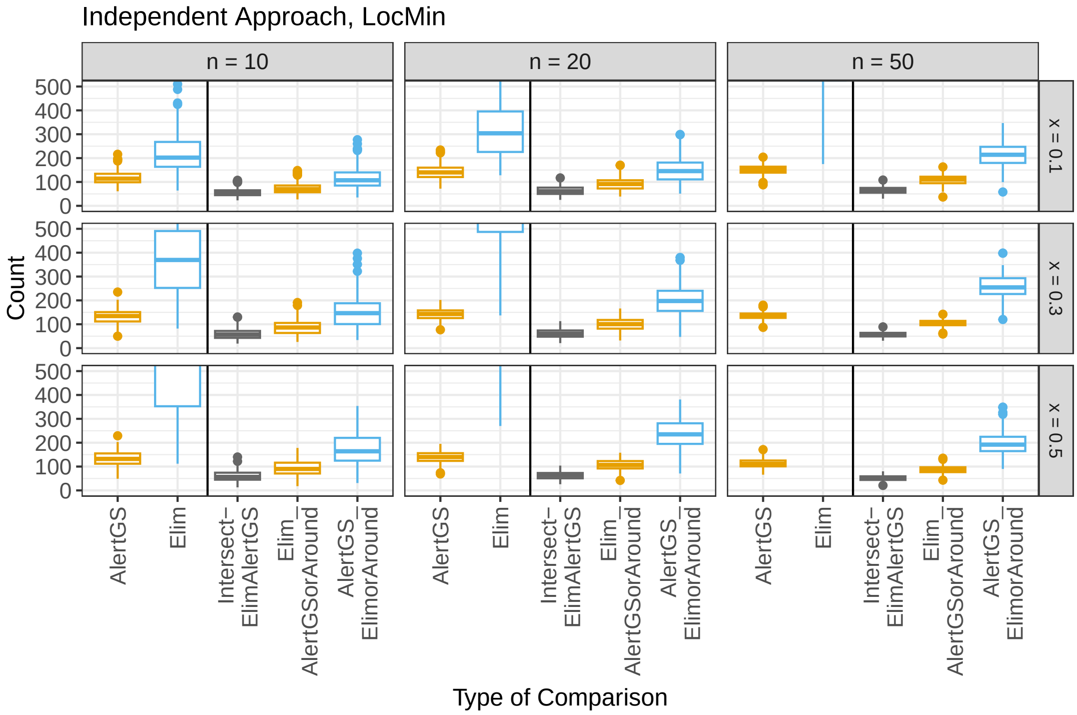

Figure C.19: The same results as shown in Figure C.18, but with a smaller y-axis limit.

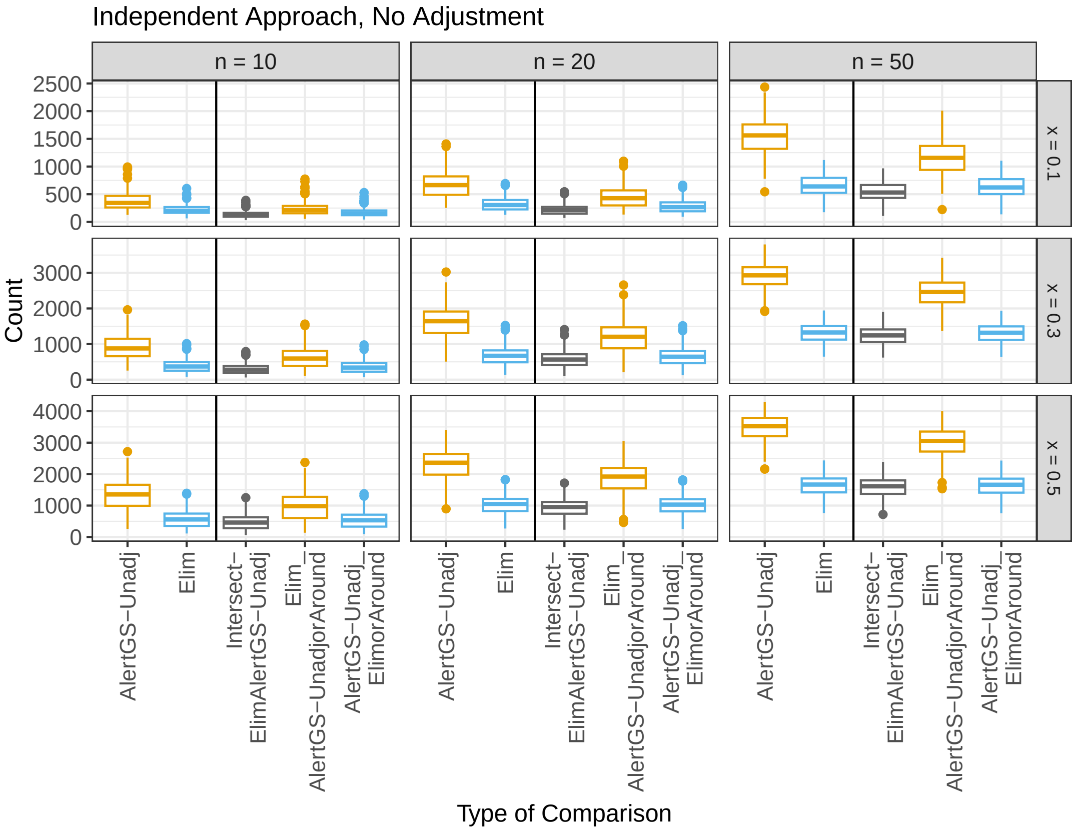

Figure C.20: Boxplots indicating the numbers of identified groups with the AlertGS (independent approach, without adjustment) and the Elim approach, as well as the number of intersections between these lists, the number of groups found by the AlertGS approach, where either the group itself or a parent/child of the group is also found by the Elim approach, and vice versa.

Comparison of the Elim approach with the true meaningful groups

Here, the results from the cutoff-based Fisher-test approach, in the 'Elim' version, to determining enriched GO groups is compared to the underlying truth. As a cutoff, 20 weeks are used. All GO groups with simulated group-wise alerts $g \leq 20$ are considered to be true groups. The identified groups with the Elim approach are compared to the true groups, and the true positive rate and the number of false positives are calculated. Since the Elim approach follows some decorrelation, instead of only considering the actually significant groups, in addition their parents and children are also considered. If not a true group itself, but any of its parents/children is found, this is considered to be a true positive. Consequently, found parents/children of true groups are not considered to be false positives.

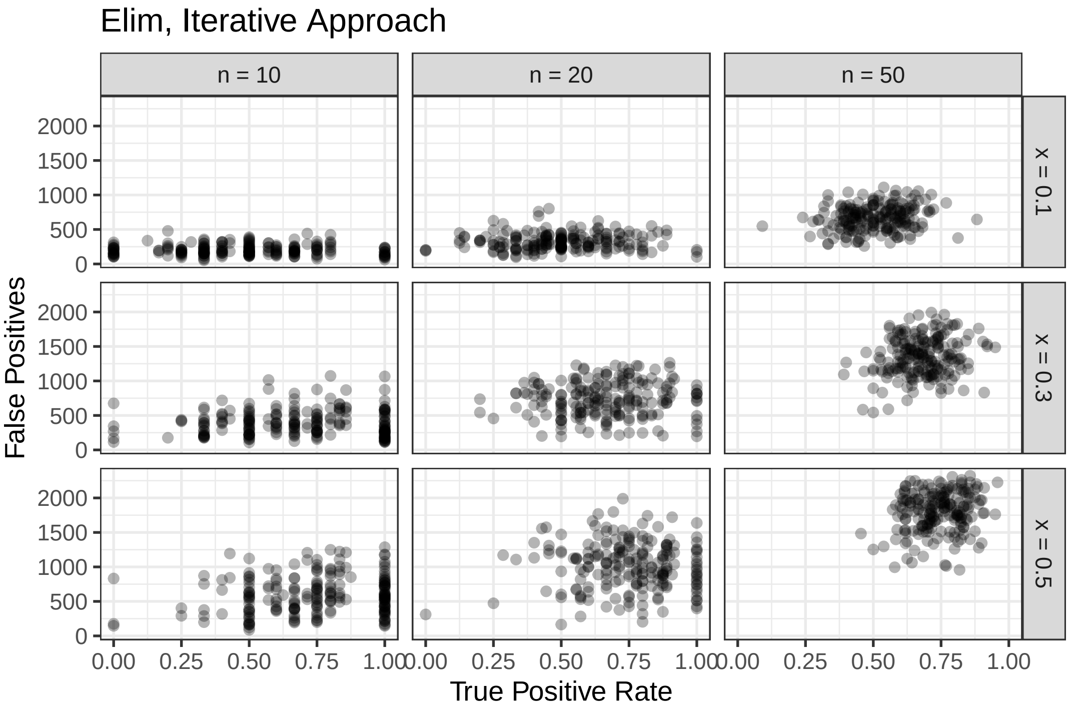

Figure C.21: Comparison of the results from the Elim-type Fisher approach with a cutoff of 20 weeks to the underlying truth, in the iterative simulation. The number of false positives is plotted against the true positive rate.

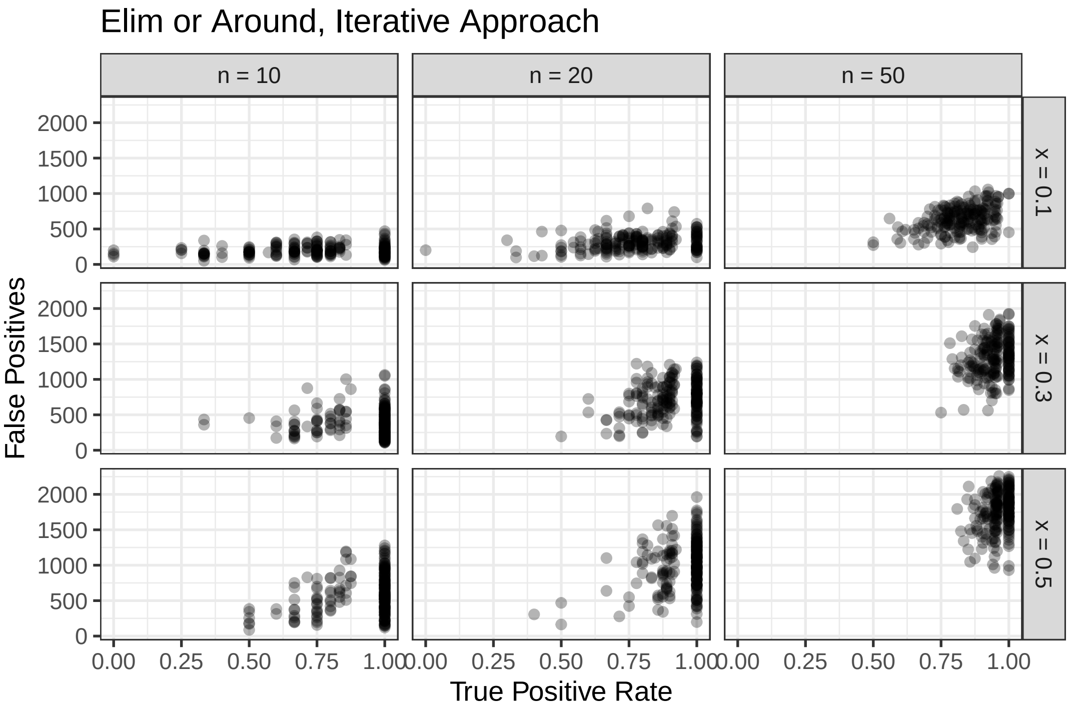

Figure C.22: Comparison of the results from the Elim-type Fisher approach with a cutoff of 20 weeks to the underlying truth, in the iterative simulation. Found parents/children of a true group are also considered to be true positives. The number of false positives is plotted against the true positive rate.

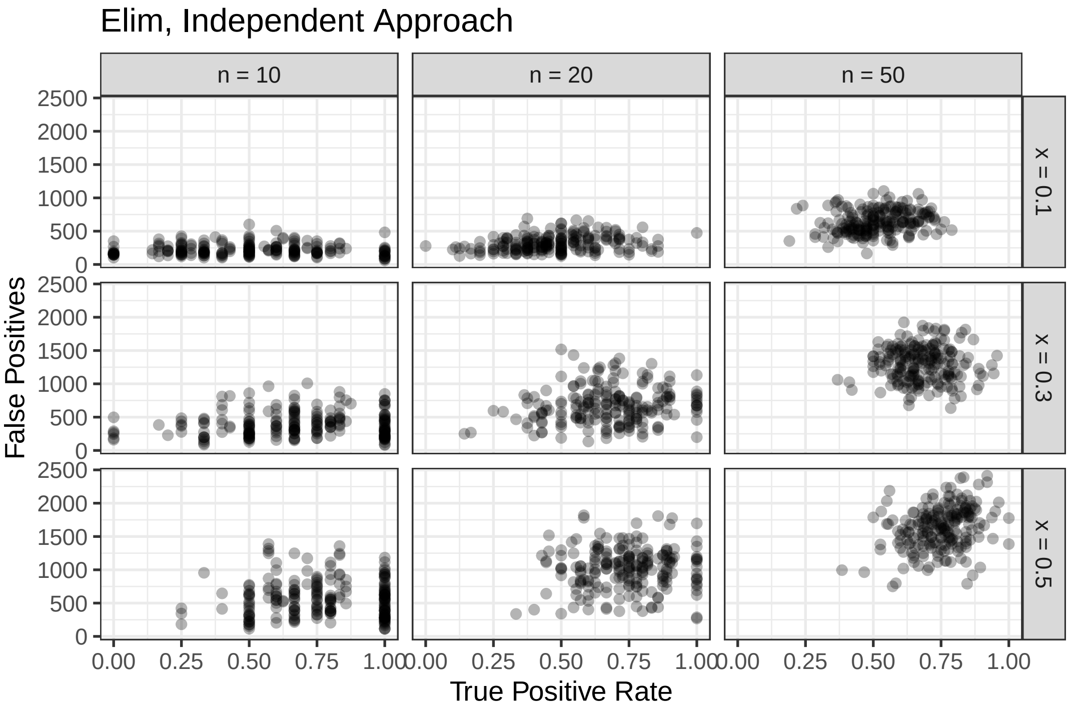

Figure C.23: Comparison of the results from the Elim-type Fisher approach with a cutoff of 20 weeks to the underlying truth, in the independent simulation. The number of false positives is plotted against the true positive rate.

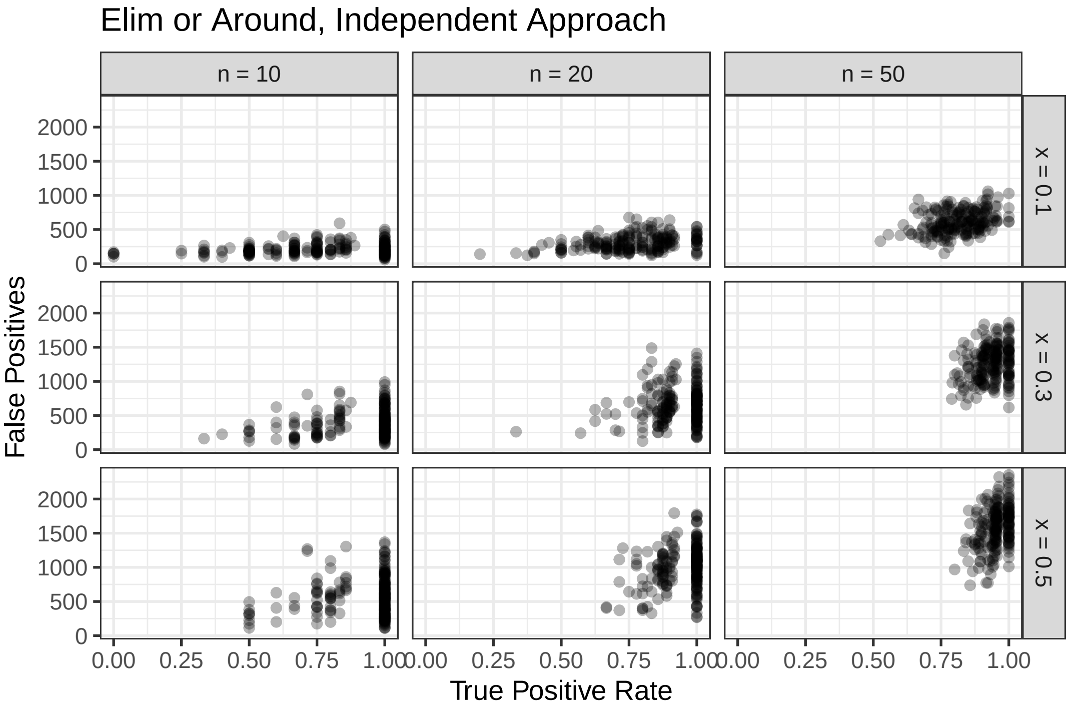

Figure C.24: Comparison of the results from the Elim-type Fisher approach with a cutoff of 20 weeks to the underlying truth, in the independent simulation. Found parents/children of a true group are also considered to be true positives. The number of false positives is plotted against the true positive rate.

Additional Results for the Case Study

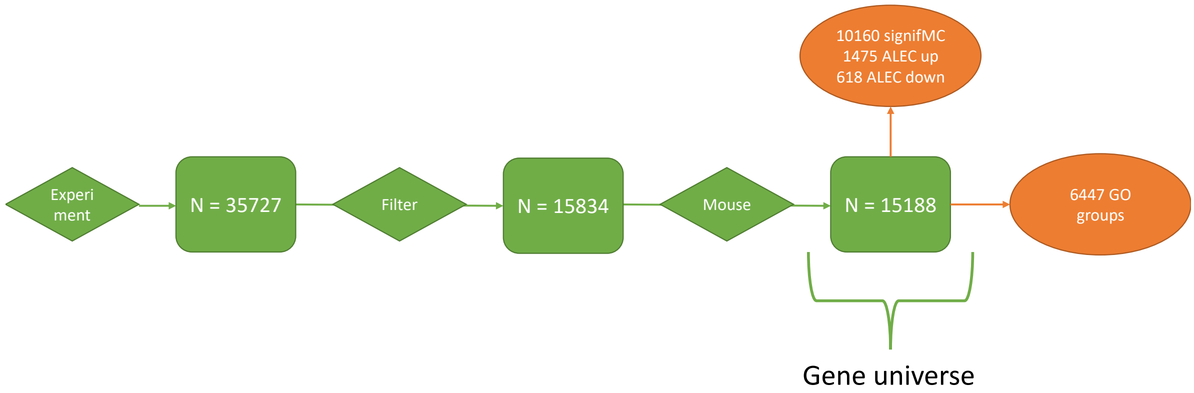

Figure C.25: Flowchart of the number of considered genes after filtering genes with too many zeros, and reducing the dataset to those genes for which annotation information for the mouse genome is available.

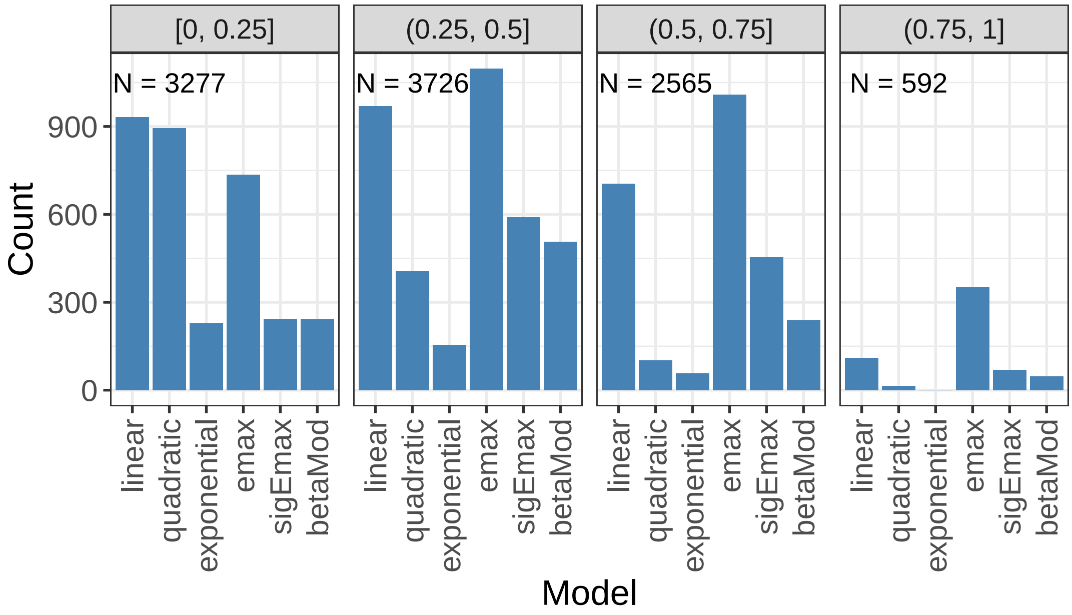

Figure C.26: Histogram showing the winner model obtained by applying the MCP-Mod approach to 15188 genes, stratified by the value of the corresponding adjusted coefficient of determination, R^2_{adj}.

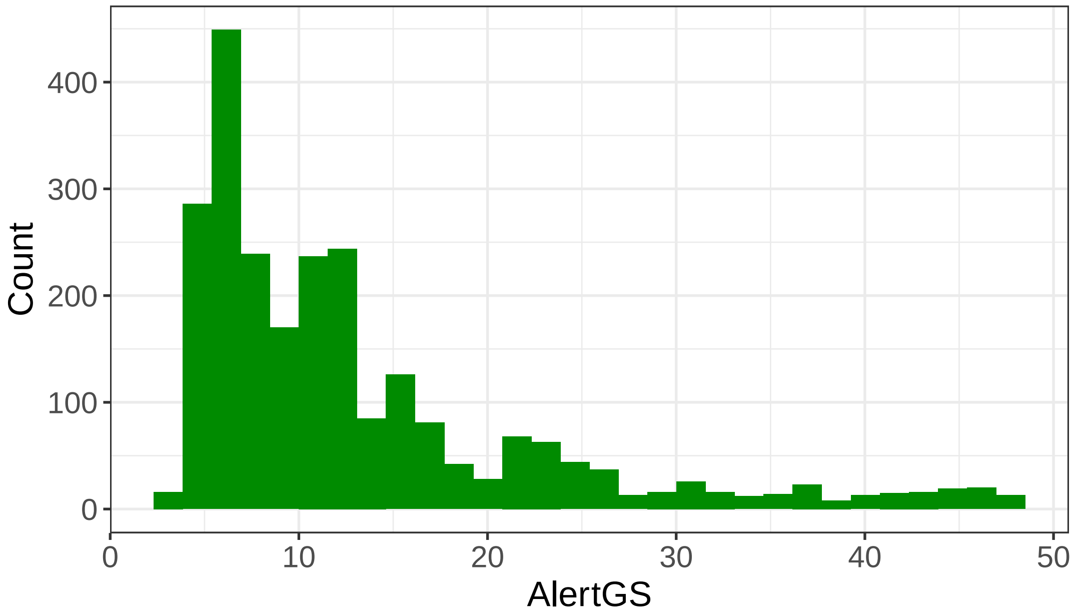

Figure C.27: Histogram of the resulting AlertGS values for all 2439 GO groups with a significant global result (i.e. a p-value smaller than or equal to 0.05), but not necessarily fulfilling the LocMin condition.

Table C.1: Overview of the identified GO groups with the Elim and the AlertGS approach, as well as the intersection of both lists (‘Intersect-ElimAlertGS’), the number of groups found by the AlertGS approach, where either the group itself or a parent/child of the group is also found by the Elim approach (‘Elim_AlertGSorAround’), and vice versa (‘AlertGS_ElimorAround‘)

Type of Comparison	Number of GO groups
Elim	904
AlertGS	152
Intersect-ElimAlertGS	67
Elim_AlertGSorAround	106
AlertGS_ElimorAround	227
