# Supplementary material for: AlertGS: determining alerts for gene sets
Source: Bioinformatics. 2025 Apr 3;41(4):btaf133. doi: 10.1093/bioinformatics/btaf133 (PMC12041417; doi:10.1093/bioinformatics/btaf133)
Supplement: btaf133_Supplementary_Data [file btaf133_supplementary_data.zip › AlertGS_SupplementB.pdf]

B Supporting Information for ‘AlertGS: Determining alerts for gene sets’: Details on the design of the simulation study

Null Situation

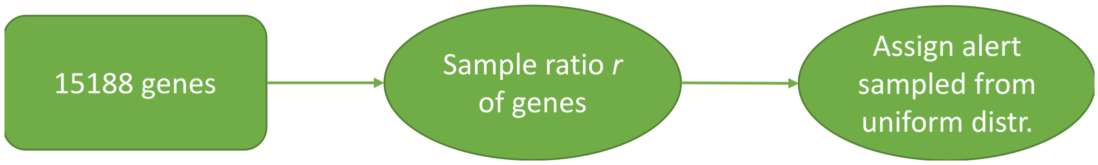

Figure B.1: Flowchart of the simulation for the null situation. The density for the uniform distribution on the interval $[3, 48]$ used in the last step is displayed in Figure B.2.

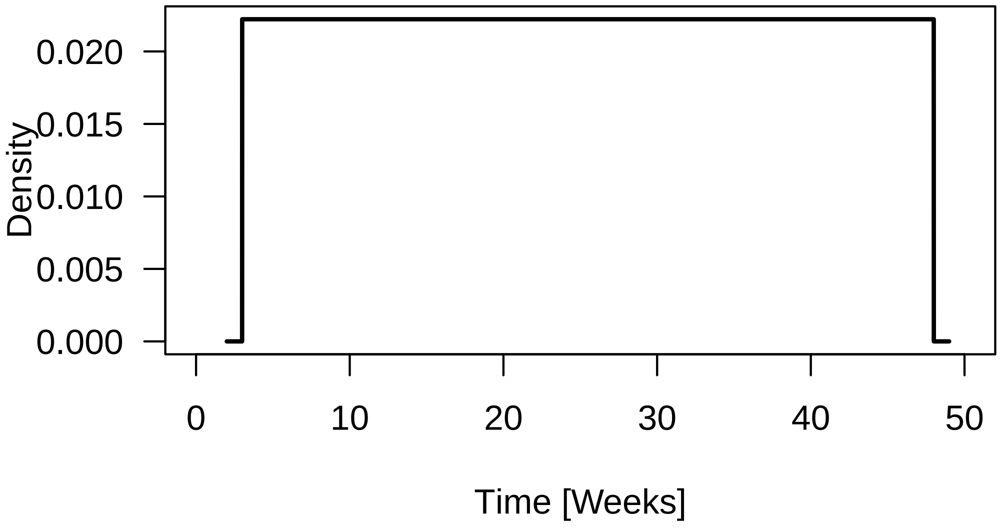

Figure B.2: Density of the uniform distribution on the interval $[3, 48]$.

Alternative Situation

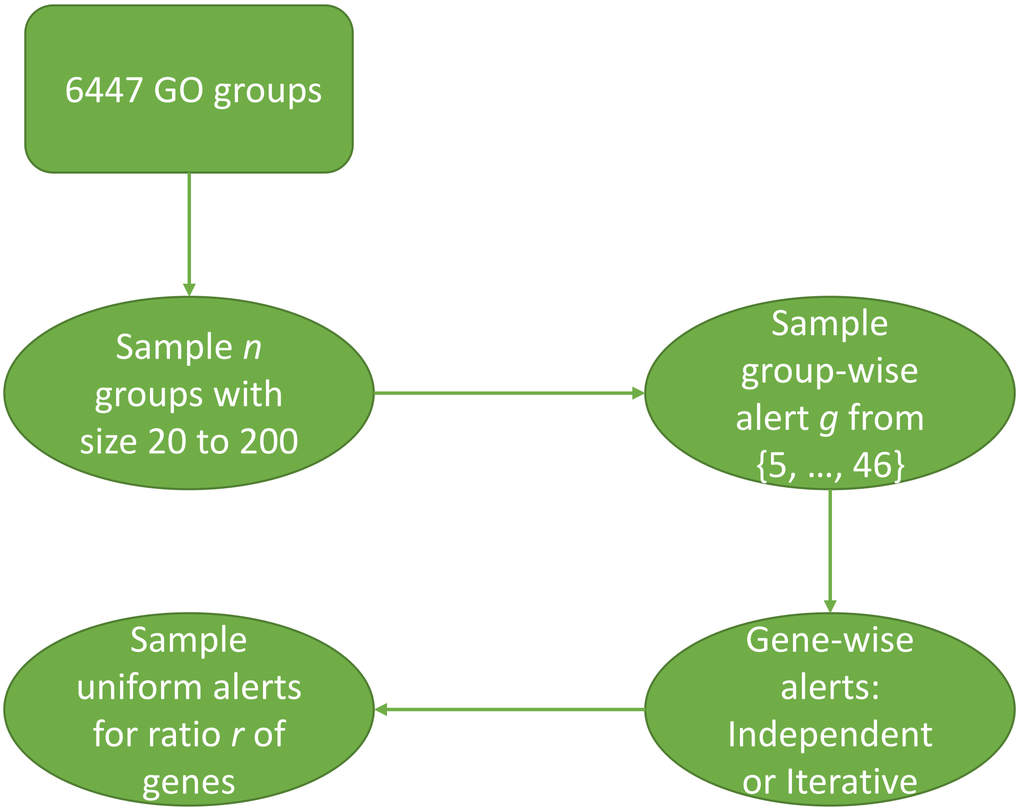

Figure B.3: Flowchart of the simulation for the alternative situation. The elements in the left column refer to the entire dataset, and the elements in the right column refer to the selected ‘meaningful’ GO groups only. More details on the two approaches *Independent* and *Iterative* are given in Figures B.5 and B.6, where the sampling of the gene-wise alerts is illustrated for one example GO group, respectively. The sampling of uniform alerts for a ratio r of genes in the last step is based on the uniform distribution again. The genes are drawn randomly from all genes, and in cases where a gene already has a sampled alert, the respective minimum of the previously sampled alert and the now uniformly drawn alert is chosen.

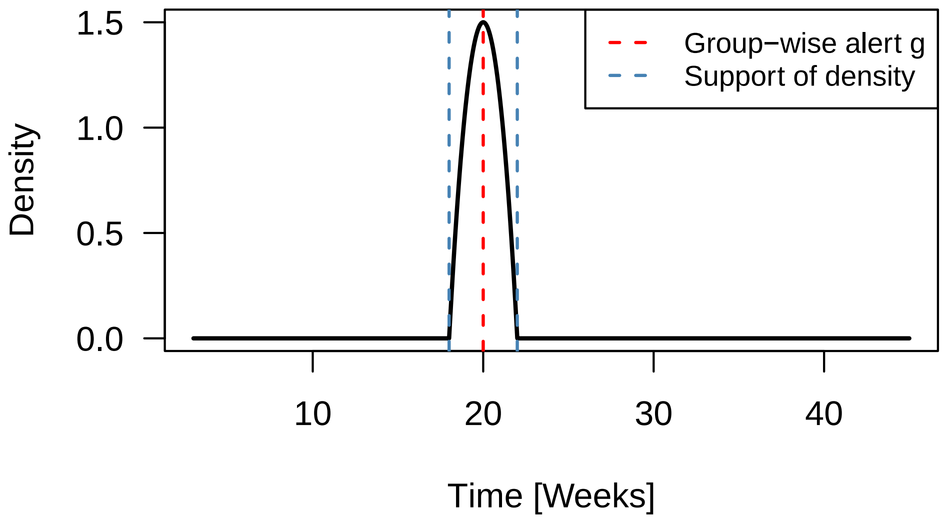

Figure B.4: Density of the shifted and scaled beta distribution with parameters $\alpha = \beta = 2$, scale parameter 4 and shift parameter $g - 2$, here for $g = 20$.

Independent Approach

	Group 1	Group 2	Group 3	Group 4	Group 5	⋯
g	32	18	20	43	9	⋯

Group 3, group-wise alert: 20

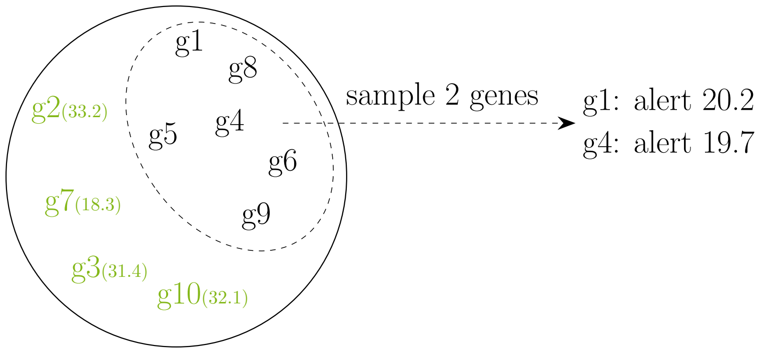

Figure B.5: Example for the independent approach. Here, group-wise alerts g of five GO groups are displayed. For Group 3 with a group-wise alert of 20, the sampling of the gene-wise alerts is sketched. It is assumed that this group consists of 10 genes. For a ratio $x = 0.2$ of genes to be sampled, this corresponds to 2 genes. Within the group, 4 genes already have an alert from previously considered groups (genes g2, g3, g7, g10, indicated in green together with the respective alert). Out of the other 6 genes without any alerts (plotted in the dashed ellipse), 2 genes are randomly drawn, and alerts are sampled based on the scaled and shifted Beta distribution with mean 20. The density of this distribution is shown in Figure B.4.

Iterative Approach

	Group 1	Group 2	Group 3	Group 4	Group 5	⋯
g	9	18	20	32	43	⋯

Group 3, group-wise alert: 20

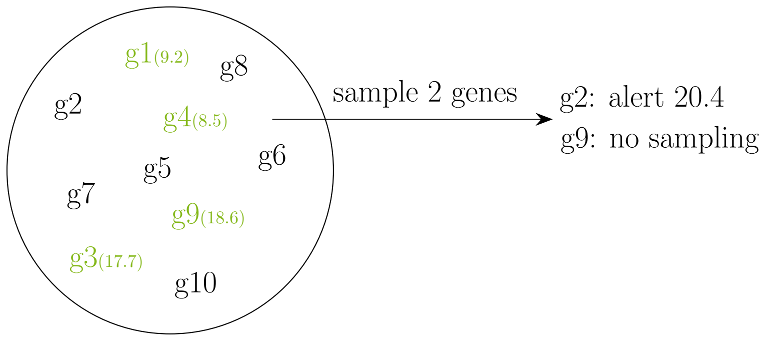

Figure B.6: Example for the iterative approach. Here, group-wise alerts g of five GO groups that are already sorted by increasing value of g are displayed. For Group 3 with a group-wise alert of 20, the sampling of the gene-wise alerts is sketched. It is assumed that this group consists of 10 genes. For a ratio $x = 0.2$ of genes to be sampled, this corresponds to 2 genes. Within the group, 4 genes already have an alert from previously considered groups (genes g1, g3, g4, g9, indicated in green together with the respective alert). The 2 genes are sampled out of all 10 genes. Here, g2 and g9 are drawn. For g2, an alert is sampled based on the scaled and shifted Beta distribution with mean 20. The density of this distribution is shown in Figure B.4. Since g9 already has an alert based on its assignment to a GO group with a lower group-wise alert, no new alert is drawn, and this gene retains the previously sampled alert.
